# Plasmablasts in previously immunologically naïve COVID-19 patients express markers indicating mucosal homing and secrete antibodies cross-reacting with SARS-CoV-2 variants and other beta-coronaviruses

**DOI:** 10.1093/cei/uxad044

**Published:** 2023-04-18

**Authors:** Anna Lundgren, Susannah Leach, Hannes Axelsson, Pauline Isakson, Kristina Nyström, Lydia Scharf, Bengt A Andersson, Nicolae Miron, Emelie Marklund, Lars-Magnus Andersson, Magnus Gisslén, Davide Angeletti, Mats Bemark

**Affiliations:** Department of Microbiology and Immunology, Sahlgrenska Academy, University of Gothenburg, Gothenburg, Sweden; Department of Clinical Immunology and Transfusion Medicine, Sahlgrenska University Hospital, Gothenburg, Sweden; Department of Microbiology and Immunology, Sahlgrenska Academy, University of Gothenburg, Gothenburg, Sweden; Department of Clinical Pharmacology, Sahlgrenska University Hospital, Gothenburg, Sweden; Department of Microbiology and Immunology, Sahlgrenska Academy, University of Gothenburg, Gothenburg, Sweden; Department of Clinical Immunology and Transfusion Medicine, Sahlgrenska University Hospital, Gothenburg, Sweden; Department of Infectious Diseases, Sahlgrenska Academy, University of Gothenburg, Gothenburg, Sweden; Department of Microbiology and Immunology, Sahlgrenska Academy, University of Gothenburg, Gothenburg, Sweden; Department of Clinical Immunology and Transfusion Medicine, Sahlgrenska University Hospital, Gothenburg, Sweden; Department of Clinical Immunology and Transfusion Medicine, Sahlgrenska University Hospital, Gothenburg, Sweden; Department of Infectious Diseases, Sahlgrenska Academy, University of Gothenburg, Gothenburg, Sweden; Department of Infectious Diseases, Sahlgrenska University Hospital, Gothenburg, Sweden; Department of Infectious Diseases, Sahlgrenska Academy, University of Gothenburg, Gothenburg, Sweden; Department of Infectious Diseases, Sahlgrenska University Hospital, Gothenburg, Sweden; Department of Infectious Diseases, Sahlgrenska Academy, University of Gothenburg, Gothenburg, Sweden; Department of Infectious Diseases, Sahlgrenska University Hospital, Gothenburg, Sweden; Department of Microbiology and Immunology, Sahlgrenska Academy, University of Gothenburg, Gothenburg, Sweden; Department of Microbiology and Immunology, Sahlgrenska Academy, University of Gothenburg, Gothenburg, Sweden; Department of Clinical Immunology and Transfusion Medicine, Sahlgrenska University Hospital, Gothenburg, Sweden

**Keywords:** COVID-19, plasmablast, Ab subclass, cross-reactivity, homing

## Abstract

Antigen-specific class-switched antibodies are detected at the same time or even before IgM in serum of non-vaccinated individuals infected with SARS-CoV-2. These derive from the first wave of plasmablasts formed. Hence, the phenotype and specificity of plasmablasts can reveal information about early B-cell activation. Here we have analyzed B cells and plasmablasts circulating in blood of COVID-19 patients not previously exposed to SARS-CoV-2 during and after disease. We find that during infection with the original Wuhan strain, plasmablasts in blood produce IgA1, IgG1, and IgM, and that most express CCR10 and integrin β1, only some integrin β7, while the majority lack CCR9. Plasmablast-secreted antibodies are reactive to the spike (S) and nucleocapsid (N) proteins of the Wuhan strain as well as later variants of concern, but also bind S proteins from endemic and non-circulating betacoronaviruses. In contrast, after recovery, antibodies produced from memory B cells target variants of SARS-CoV-2 and SARS-CoV-1 but compared to previously non-infected individuals do not show increased binding to endemic coronaviruses. This suggests that the early antibody response to a large extent stems from pre-existing cross-reactive class-switched memory B cells, and that although newly formed memory cells target the novel SARS-CoV-2 virus the numbers of broadly cross-reactive memory B cells do not increase extensively. The observations give insight into the role of pre-existing memory B cells in early antibody responses to novel pathogens and may explain why class-switched antibodies are detected early in the serum of COVID-19 patients.

## Teaser

During an infection, plasmablasts circulating in blood represent ongoing formation of antibody-producing cells from activated B cells. Here we study the early plasmablasts in previously naïve COVID-19 patients arriving at hospital. We find extensive cross-reactivity to circulating and non-circulating beta-coronaviruses, that IgA1 responses dominate, and that the cells express markers suggesting mucosal homing.

## Introduction

The betacoronavirus (beta-CoV) SARS-CoV-2 that causes COVID-19 was identified in humans early in 2020 and resulted in a world-wide pandemic that is still causing morbidity and mortality in 2023. The virus mainly spreads via droplets and aerosols and has gradually developed into a highly contagiou`s pathogen [1–5]. Most infected individuals successfully clear the infection after activation of the adaptive immune system [6, 7]. Neutralizing antibodies (Abs) and antigen-specific CD4 and CD8 cells form, and immunity and immunological memory subsequently develop [6, 7]. Class-switched Abs against the virus can be detected in serum at the same time or even before IgM when previously naïve individuals are infected [8, 9], and IgA may contribute significantly to blocking viral infectivity early in the response [10].

However, levels of serum Abs do not directly reflect the strength and functional characteristics of B cell responses. Depending on the site of B cell induction, distinct differentiation pathways are triggered, organ-specific cues ensure that plasmablasts (PBs) secrete appropriate Ab classes and home to the optimal effector sites before they mature into effector plasma cells (PCs) [11]. Homing of the PBs is mediated by the expression of integrins and chemokine receptors. Long-term humoral immunity is subsequently ensured through a combination of Abs produced by long-lived PCs at effector sites and the long-term survival of specific memory B cells that can rapidly replenish the PC pool after activation [12].

There is currently no coherent picture of the early B-cell response in previously immunologically naïve COVID-19 patients. Some studies have suggested that germinal center (GC) formation may be inhibited during disease and that responding B cells are primarily activated in T-independent extrafollicular responses [13, 14]. Others imply that class-switched Abs and memory B cells are present already early in the response, features that would normally be associated with T-cell dependent responses and GC formation [7, 8, 15, 16]. The most important antigen for Ab-mediated protection against coronavirus infection is the spike (S) protein that binds specific receptors which mediate viral entry into cells during infection. The S protein shows considerable sequence similarity between SARS-CoV-1 and SARS-CoV-2, both using angiotensin converting enzyme-2 (ACE-2) as their receptor, but other coronaviruses show larger diversity and, with the exception of the endemic alphacoronavirus NL63, bind other receptors [17, 18]. Nonetheless, T cells and serum Abs reactive against the S protein of SARS-CoV-2 have been detected in samples collected even before the pandemic [19–23], and an increased production of cross-CoV-reactive Abs has been observed after infection with SARS-CoV-2 [24, 25].

Preexisting memory B cells can influence the response to novel but related pathogens [26]. The most well-known example is influenza type A, for which memory B cells formed during the very first infection will influence all subsequent immune responses to type A influenza strains [27]. During the pandemic, SARS-CoV-2 variants of concerns (VOC), displaying ever increasing abilities to escape serum Ab neutralization, have developed from the original Wuhan strain [2, 4]. Still, immunological memory hinders severe morbidity and mortality, and the previous history of vaccinations and infections influences the response to new variants, suggesting a similar mechanism for protection against SARS-CoV-2 VOC as for different influenza strains [28, 29]. Despite the large diversity in the S regions of beta-CoVs, cross-reactive memory B cells have been suggested to play a role in the first response to SARS-CoV-2 antigens [24, 25, 30], but the relative importance of such activation has, as far as we are aware, not been evaluated in detail in previously non-infected and non-vaccinated COVID-19 patients.

Here we have studied B cell responses in immunologically naïve patients infected by the original Wuhan SARS-CoV-2 strain early in the pandemic before any vaccines were available. We find that these patients had developed strong PB responses 4–24 days after their first signs of disease and that the early PBs produced IgA1 and expressed receptors suggestive of mucosal, but not gut, homing. Class-switched PBs also showed significant cross-reactivity to previously encountered beta-CoVs, but also to SARS-CoV-2 VOC yet to develop as well as to related non-circulating strains. The study gives insights into the early B cell response against SARS-CoV-2 during COVID-19 infection and how it is influenced by previous coronavirus exposures. It supports the notion that the early PB responses may, at least partly, be derived from cross-reactive pre-existing memory B cells.

## Materials and methods

### Study participants, healthy controls, and blood sampling

Patients (*n* = 47) admitted to the Infectious Diseases or Intensive Care Units at the Sahlgrenska University Hospital between 27 April and 9 June 2020 with PCR-confirmed SARS-CoV-2 infection were enrolled in the study. Two non-hospitalized subjects with PCR-confirmed infection were also included (total *n* = 49 patients, Table 1, Supplementary Fig. S1).

**Table 1.** Demographics, clinical characteristics, and sampling of COVID-19 infected patients in the study

	Demographics Covid patients (*n* = 49)	Demographics Recovered patients (*n* = 20)^a^
Age, years	54 (27–77)	54 (31–67)
Sex		
Male	36 (73%)	16 (80%)
Female	13 (27%)	4 (20%)
	
Fever (>38°C)	44 (90%)	19 (95%)
Cough	37 (76%)	19 (95%)
Low blood oxygen saturation (SpO_2_ <94%)	42 (86%)	18 (90%)
Dyspnoea	23 (47%)	17 (85%)
Headache	8 (16%)	6 (30%)
Nausea	6 (12%)	4 (20%)
Diarrhoea	4 (8%)	3 (15%)
Hospitalization		
Required inpatient care	47 (96%)	20 (100%)
Duration of hospitalization, days	10 (2–167)	8 (3–21)
Sampling		
Symptom onset to sampling, days	11 (4–24)	10 (5–16)
Hospitalization to sampling, days	2 (0–20)	2 (0–8)
Oxygen treatment on day of sampling		
Supplemental oxygen	42 (86%)	18 (90%)
High-flow nasal oxygen	11 (22%)	8 (40%)
Mechanical ventilation	14 (29%)	1 (5%)
Disease severity		
Mild	2 (4%)	0 (0%)
Moderate	5 (10%)	3 (15%)
Severe	27 (55%)	16 (80%)
Critical	15 (31%)	1 (5%)
Deceased	2 (4%)	0 (0%)
Chronic comorbidities		
Hypertension	14 (29%)	5 (25%)
Diabetes (types 1 and 2)	10 (20%)	4 (20%)
Chronic heart disease	4 (8%)	0 (0%)
Chronic lung disease	6 (12%)	4 (20%)
Chronic kidney disease	2 (4%)	0 (0%)
Malignancy	2 (4%)	0 (0%)
Immunosuppression	2 (4%)	1 (5%)
None	15 (31%)	10 (50%)
**≥** 1 comorbidity	28 (57%)	7 (35%)
**≥** 2 comorbidity	9 (18%)	3 (15%)
**≥** 3 comorbidity	3 (6%)	0 (0%)

^a^All individuals in the group “recovered patients” were also part of the “Covid patients” group. Data are presented as number of patients and % or median range within brackets.

Blood samples were collected as soon after hospital admission as possible (median 2 days) giving a median of 11 days after first symptoms (range 4–24 days; Table 1). The majority (73%) of the enrolled patients were males and the median age was 54 years. Disease severity was classified as mild, moderate, severe, and critical, as defined by the COVID-19 Treatment Guidelines Panel of the National Institutes of Health [31]. The disease severity ranged from mild in the non-hospitalized patients (4%), to moderate (10%), severe (55%), or critical (31%) in the hospitalized patients (Table 1). From 14 of the 47 subjects with moderate to critical disease, samples were also collected approximately 3 months (median 92 days, range 70–105) after collection of the first sample (recovered patients). In addition, samples were collected from 10 of the patients with moderate to critical disease approximately 7 months (median 218 days, range 183–227 days) after the first sampling for memory analyses. Samples collected from 10 healthcare workers (68% females, median 39 years, range 35–63 years) without COVID-19 disease (no positive COVID-19 PCR test and no COVID-19 seroconversion before sample collection) were enrolled as controls for the memory B cell analyses, and samples from five other health care workers were used as healthy controls for flow cytometry.

For the clinical flow cytometric panels, data collected from 61 healthy blood donors before the pandemic were used as controls. Age and sex of these donors had been anonymized before samples arrived at the laboratory, but they are random samples from an overall pool of blood donations that originated from donors with a median age between 45 and 50 years of age and was to 59% from male donors. Frozen serum, plasma, and Abs secreted into lymphocyte supernatants (ALS) samples collected from 19 healthy subjects (68% males, median 41 years, range 25–71 years) enrolled in two other studies performed at the University of Gothenburg and Sahlgrenska University Hospital before the pandemic were used as additional negative controls. Subjects enrolled in these studies had approved the use of collected samples as controls in other immunological studies after sample anonymization.

### Sample collection and preparation

Blood was collected in EDTA or Lithium-Heparin tubes and samples were processed for analysis within 24 h after collection. Samples collected in EDTA tubes were either used directly to determine the concentrations of T, B, and NK cells or were washed twice using BD FACSFlow Sheath Fluid (BD Biosciences, San Jose, CA) or eBioscience Flow Cytometry Staining Buffer (ThermoFisher Scientific, Waltham, MA) by repeated centrifugation at 200 x *g* to remove serum Abs. After washing, the cells were resuspended in washing buffer to the same volume as the original blood sample.

Samples collected in lithium heparin tubes were diluted 1:2 in PBS, and PBMCs were isolated through density gradient centrifugation using Lymphoprep in accordance with the instructions from the manufacturer (Alere Technologies AS, Oslo, Norway). Plasma diluted 1:2 was collected from supernatants after the first centrifugation step and stored at −70°C until Ab analysis. Serum samples collected in serum tubes were used for control purposes. PBMCs were frozen in fetal calf serum (Sigma, 50%), RPMI (Lonza, Basel, Switzerland; 40%), and DMSO (Sigma, 10%) and kept in 1 ml aliquots at −135 °C until analysis.

For analysis of Abs secreted from PBs (Antibody secreting cells; ASCs) using the ALS method, fresh PBMCs were cultured in RPMI 1640 culture medium (Lonza) supplemented with 10% fetal calf serum (Sigma), gentamicin (100 µg/ml, Sigma), mercaptoethanol (50 µM, Sigma), and l-glutamine (0.3 mg/ml, Sigma) in a 37 °C incubator with 5% CO_2_. PBMCs were cultured in flat-bottomed 96-well plates (2 × 10^6^ cells per 200 µl/well) without stimuli. Supernatants were collected after 48 h, and stored at −70 °C until Ab analysis. For analysis of memory B cells, 2 × 10^6^ frozen PBMCs were cultured in 2 ml/well in 24-well plates and stimulated with 1 µg/ml R848 and 10 ng/ml IL-2 (Both from Mabtech, Stockholm, Sweden). Supernatants were collected after 9 days, centrifuged at 300 x *g* for 5 min and stored at −70 °C until Ab analysis.

### Flow cytometry

All flow cytometry was performed using fresh blood samples collected in EDTA tubes within 24 h of collection. Concentrations of T, B, and NK cells were determined in 50 µl of non-processed blood using BD multitest 6-color TBNK reagent with Trucount tubes in accordance with the instructions from the manufacturer (BD Biosciences). Cells were incubated with Abs for 20 min after which 450 µl of 1 × BD FACS Lysing solution was added and the cells were incubated for at least 15 min before analysis. Samples were analyzed within 3 h after staining using BD FACSCanto flow cytometers.

The clinical assay for determination of B cell subsets was performed using the Abs listed in Supplementary Table S1. Flow cytometry Abs were mixed with 100 µl of blood washed as described above to remove serum Abs. After incubation at room temperature for 20 min, erythrocytes were lysed using 1 × BD FACS lysing solution, and the cells were subsequently washed using a BD FACS Lyse Wash Assistant. The relative proportion of cells belonging to different B-cell subsets among CD19^+^ cells was determined using FACSCanto flow cytometers. For calculating cell concentrations, the relative proportion of cells within a subset was multiplied by the total concentration of CD19^+^ B cells. Normal concentrations were determined from a group of 31 healthy blood donors analyzed before the pandemic; clinical results are rounded to the closest 0.01 × 10^6^ cells/ml. For comparison of relative proportions, data from patients were compared to data from 61 healthy blood donors collected before the pandemic. Data were analyzed using FACSDiva (BD Bioscience) exactly as done for clinical samples (Supplementary Fig. S2B).

Expression of homing markers or memory cell markers were analyzed using the same protocol as above but with the addition of Brilliant Stain Buffer Plus (BD Biosciences) to avoid interactions between Brilliant Violet Abs (Abs used listed in Supplementary Table S2). When isotype expression was determined, extracellular Abs were first detected as above, but after 20 min, 2 ml of 1 × RBC Lysis buffer (Thermo Fisher Scientific) was added, and after 10 min cells were pelleted using centrifugation at 400 x *g*. After washing the cells twice using Flow Cytometry Staining Buffer (Thermo Fisher Scientific), the cells were fixed and permeabilized using BD Cytofix/Cytoperm solution in accordance with the instructions from the manufacturer (BD Bioscience), after which Abs targeting intracellular isotypes were added. After a 30 min incubation at room temperature in the dark, the cells were washed twice with Cytoperm solution. For detection of T follicular helper (Tfh) cells, 100 µl of blood were incubated with Abs against extracellular antigens and erythrocytes lysed, cells fixed, permeabilized, and stained with anti-FoxP3 Ab as described in eBioscience Human Regulatory T cells whole blood staining kit (Thermo Fisher Scientific). Analysis was performed using FlowJo 9.9.6 as outlined in Supplementary Figs. S2, S3, S5, and S6.

### Ab analyses

Levels of IgM, IgG, and IgA Abs in plasma, serum, ALS specimens, and samples derived from cultures of stimulated memory B cells were determined using multiplex electrochemiluminiscence (ECL) assays including SARS-CoV-2 Spike (S), Receptor binding domain (RBD), N-terminal domain (NTD) and Nucleocapsid (N) antigens from the original Wuhan strain as well as SARS-CoV-1, MERS, OC43, and HKU S antigens and Influenza H3 antigen (Corona Virus panel 1; K15362U-K15364U) or SARS-CoV-2 variant panel including the original Wuhan strain and S proteins from Alpha, Beta, Gamma, Delta, and Omicron variants (Corona Virus panel 23; K15567U and K11569U) analyzed on a Meso Quickplex SQ 120 reader (Meso Scale Discovery, Rockville, USA). Antigen details can be found in Supplementary Table S3. Serum and plasma samples were analyzed at a 1/5000 dilution, according to the manufacturers’ instructions, ALS samples were analyzed at a 1/10 dilution and memory B cell samples at 1/10 (IgM, IgG, and IgA) and 1/1000 (IgG) dilution.

### Statistics

All statistics were calculated using Prism software (Graphpad, San Diego, CA). All statistical comparisons between groups and correlations were calculated using nonparametric methods (Mann–Whitney with Holms–Bonferroni correction when applicable, Kruskal–Wallis followed by Dunn’s multiple comparisons test, or Spearman rank correlation) assuming non-paired relationships between samples. Wilcoxon matched pairs signed rank test was used to compare paired samples. All comparisons giving *P*-values less than 0.05 are indicated by values rounded off to one significant figure. All graphs show samples from different individuals as symbols and the median of all included samples as bars.

## Results

### Strong plasmablast responses in COVID-19 patients during acute infection

**Figure 1.**
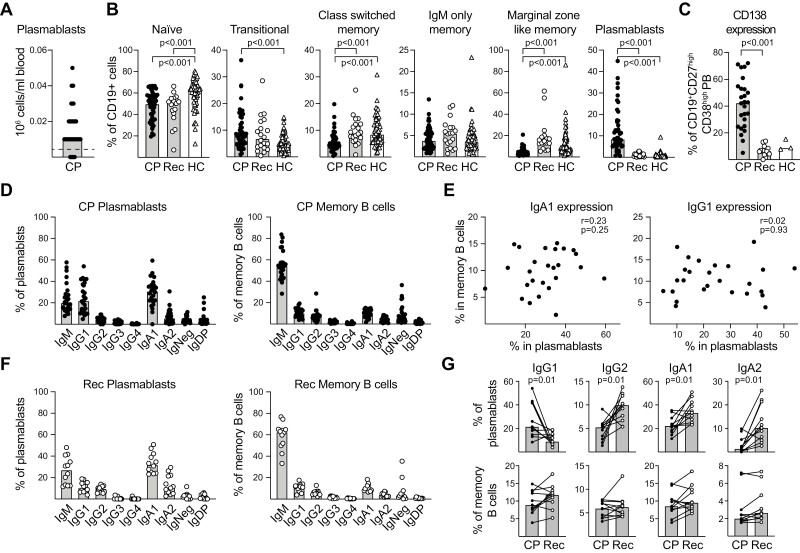
IgA1, IgG1, and IgM plasmablasts dominate the early response to SARS-CoV-2 in COVID-19 patients. (**A**) The concentration of blood PBs, rounded to the closest 0.01 × 10^6^ cells, was determined in COVID-19 patients (CP) using a clinically validated flow cytometric assay. The dashed line indicates the value that 95% of healthy blood donors are below. (**B**) The proportion of CD19^+^ cells belonging to the indicated B-cell subtypes compared between acutely infected COVID-19 patients (*n* = 49), patients 3 months after recovery (Rec, *n* = 20), and healthy pre-pandemic blood donors (HC, *n* = 61). (**C**) The proportion of PBs that express CD138 in CP (*n* = 26), Rec (*n* = 12), and HC (*n* = 3). (**D**) The proportion of PBs and memory B cells that express the indicated Ab subclasses from CP (*n* = 26) and (**E**) correlation plots comparing the expression of IgA1 and IgG1 on memory cells and PBs in individual patients. (**F**) Ab subclass expression on PBs and memory B cells from recovered patients (*n* = 12) presented as in (D). (**G**) Changes in the proportion of cells that expressed different Ab subclasses from matched CP and Rec samples from 11 patients for which data from both time points was available. (A-G) Individual values are marked with black dots (CP), white dots (Rec), or white triangles (HC) with bars indicating group medians. Statistical comparisons between groups and correlations were calculated using nonparametric methods (Mann–Whitney, Kruskal–Wallis followed by Dunn’s multiple comparisons test, Wilcoxon matched pairs signed rank test, and Spearman rank correlation); only differences with *P* < 0.05 are indicated. Gating strategies and additional data can be found in Supplementary Fig. S2.

The concentrations of cells belonging to major lymphoid lineages were determined in blood collected from COVID-19 patients close after arrival at the Sahlgrenska University Hospital in Sweden (*n* = 49; covid patients; CP) or approximately 2–3 months after they had recovered (*n* = 20; Rec) (Table 1, Supplementary Fig. S1). Among these, 47 were hospitalized with moderate to severe disease, while two did not require hospital care. The initial samples were collected early during the pandemic (April–June 2020) when the Wuhan strain dominated, reinfections were unlikely, and before any vaccines were available. In line with previous studies, T-cell lymphopenia was common in patients [7]; more than half had concentrations of lymphocytes, CD3^+^ and CD3^+^CD8^+^ T cells below normal clinical ranges defined in healthy blood donors before the pandemic, and more than a quarter had sub-normal concentrations of CD3^+^CD4^+^ T cells (Table 2, Supplementary Fig. S2A). The concentrations of CD19^+^ B cells were below normal levels in 33% of the patients (Table 2, Supplementary Fig. S2A). All B cell subtypes measured had lowered concentrations in blood, the exceptions being transitional B cells, for which levels both above and below the normal range were recorded, and PBs, for which increased concentrations were found in 86% (42/49) of the patients (Table 2, Fig. 1A and Supplemental Fig. S2B, S2C). Concentrations of all cell types in blood increased 2–3 months after recovery, with previously infected patients having normal or even increased levels of cells (Table 2, Supplementary Fig. S2).

**Table 2.** Proportions of COVID-19 patients with concentrations of different lymphocytes and B-cell subsets above or below the normal range in healthy blood donors

	Clinical normal range (10^6^ cells/ml)	Proportions of CP with cell concentrations below the normal range	Proportions of CP with cell concentrations above the normal range	Proportions of Rec with cell concentrations below the normal range	Proportions of Rec with cell concentrations above the normal range
T B and NK lymphocytes					
Lymphocytes	1.0–2.8	25/49 (51%)			3/20 (15%)
T cells	0.7–2.1	25/49 (51%)			3/20 (15%)
CD4+ T cells	0.3–1.4	13/49 (27%)			4/20 (20%)
CD8+ T cells	0.2–0.9	27/49 (55%)		2/20 (10%)	2/20 (10%)
NK cells	0.09–0.6	14/49 (29%)			
B cells	0.1–0.5	16/49 (33%)			1/20 (5%)
B cell subtypes					
Naïve cells	0.08–0.14	28/49 (57%)	4/49 (8%)	5/20 (25%)	7/20 (35%)
Transitional cells	0.01	13/49 (27%)	19/49 (39%)	3/20 (15%)	12/20 (60%)
Class-switched memory cells	0.01–0.03	20/49 (41%)		1/20 (5%)	5/20 (25%)
Marginal zone-like memory cells	0.01–0.03	26/49 (53%)			11/20 (55%)
IgM+ memory cells	0.01	24/49 (49%)	2/49 (4%)	2/20 (10%)	8/20 (40%)
Plasma blasts	<0.01		42/49 (86%)		2/20 (10%)

In the table CP = Covid Patient with active disease and Rec = Recovered Covid Patient three months after infection.

When the proportions of B cells belonging to different subtypes were compared, PB frequencies were significantly higher in CP than in healthy blood donors (HC) or Rec (Fig. 1B). An increased proportion of transitional B cells was evident during disease, while mature naïve cells were reduced (Fig. 1B). The proportion of class-switched and IgM^+^IgD^+^ marginal zone-like CD27^+^ memory cells were decreased while the proportion of IgM only memory cells was not significantly different from that in HC. After recovery, the proportions of B cell subtypes in blood normalized to values comparable to those in HC, the exception being naïve cells that were still lowered (Fig. 1B).

### The COVID-19 plasmablast response is dominated by cells expressing IgA1 or IgG1 and mucosal homing markers

The high levels of PBs in blood of CP allowed us to phenotypically characterize these cells that were likely formed in an antigen-specific response against the Wuhan strain of SARS-CoV-2. Hence, PBs from fresh blood were analyzed from 26 CP and compared to those from 12 Rec (11/12 subjects also included in the CP group) and three (out of five) HC that had detectable PB populations in blood. High expression of CD138 is associated with later stages of PB towards PC development and possibly plays a role in long-term survival of PCs [32]. In CP, a median of 42.5% of the PBs expressed CD138, while the proportion of PBs expressing high levels of CD138 was lower in Rec and HC (Fig. 1C). In fact, the proportion of PBs that expressed high levels of the marker did not exceed 15% in any of the Rec or HC but it did so in 18 out of 20 CP.

Next, we used a set of monoclonal Abs able to distinguish IgA and IgG subclasses [33] together with Abs against IgM and IgD to determine the Ab isotypes and subclasses expressed by different B-cell subsets. In CP, the most common subclass produced by PBs was IgA1, with substantial numbers of cells instead expressing IgG1 or IgM (Fig. 1D, Supplementary Fig. S2D, S2E). Among CD27^+^ memory B cells from CP, IgM expressing cells dominated with IgG1 and IgA1 being the most common switched subclasses (Fig. 1D). There was no correlation between the proportions of cells that expressed IgA1 or IgG1 in PBs and memory B cells, arguing against extensive polyclonal PB formation from bystander non-specific memory B cells (Fig. 1E). The proportion of PBs expressing IgG1 was lower in Rec than in CP while the proportions of IgG2^+^, IgA1^+^, and IgA2^+^ cells were higher, likely reflecting that human gut derived PBs that recognize diverse antigens present in the gut dominate at steady state (Fig. 1F and G) [34].

**Figure 2.**
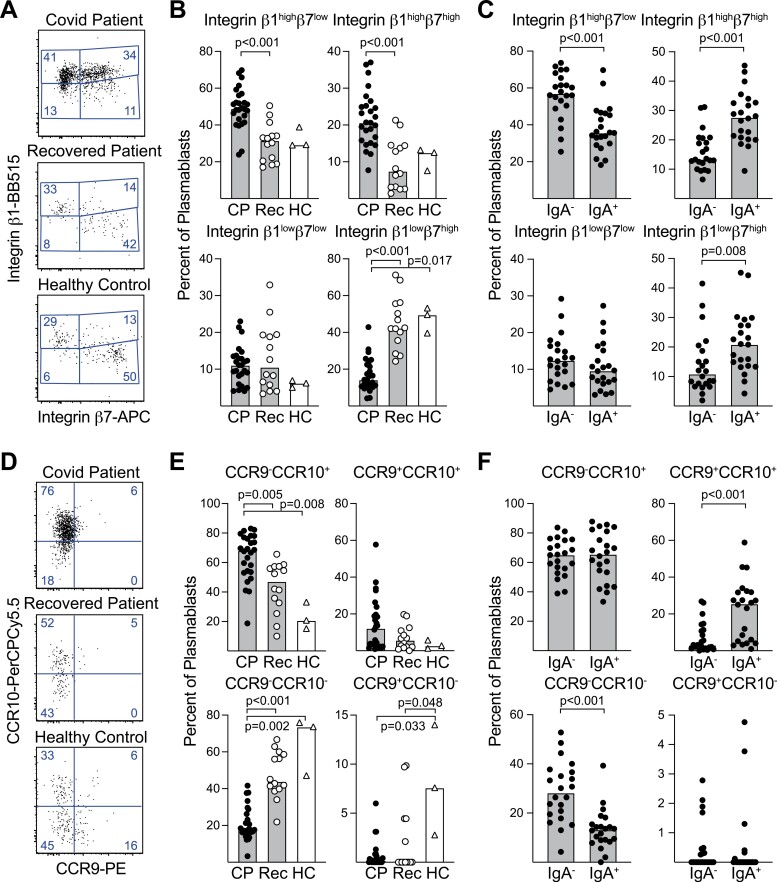
Plasmablasts in COVID-19 patients express high levels of integrin β1 and CCR10, variable levels of integrin β7 and little CCR9. The expression of homing markers was determined on PBs (CD19^+^CD38^high^CD27^high^) by flow cytometry. (**A**) PBs were divided into quadrants based on the expression of integrin β1 and β7, and (**B**) the proportion of cells in each quadrant was determined in COVID-19 patients (CP; *n* = 26), recovered COVID-19 patients (Rec; *n* = 14) and healthy controls that had sufficient numbers of PBs for analysis (HC; *n* =3 of five tested). (**C**) PBs from CP were split based on surface IgA expression and analyzed as in (A). (**D–F**) Expression of CCR10 and CCR9 on PBs determined as in (A-C). Individual values are indicated by symbols and group medians by bars. Statistical comparisons between groups were calculated using Mann–Whitney (C and F) or Kruskal–Wallis followed by Dunn’s multiple comparisons test (B and E); *P* values < 0.05 are indicated. Gating strategies, controls, and additional data can be found in Supplementary Fig. S3.

Organ-specific cues determine which homing receptors will be induced and consequently to which tissue a PB will migrate [11]. PB expression of the chemokine receptor CCR10 and the integrin pair α4β1 has been associated with cells migrating into mucosal tissues, whereas co-expression of these markers together with CCR9 and/or integrin α4β7 specifically targets cells to the gut. Expression of CD62L or CXCR4 is associated with homing to bone marrow or inflamed tissues, respectively, particularly for cells lacking mucosal homing receptors [35]. PBs from CP expressed higher levels of integrin β1, CCR9, CCR10, and CD62L than other B cell subtypes, but similar levels of integrin β7 and lower levels of CXCR4, as judged by their median fluorescent index (Supplementary Fig. S3A and S3B). When the proportion of PBs expressing these homing markers were compared among CP, Rec, and HC, most PBs from CP expressed integrin β1 (a median of 71.1%) while this homing marker was present on less than half of the cells in Rec and HC (39.9% and 43.5%, respectively) (Fig. 2A and B). Regardless of disease state, approximately a third of PBs expressing integrin β1 co-expressed integrin β7. PBs that lacked integrin β1 dominated in Rec and HC, and most of these expressed high levels of integrin β7. PBs lacking integrin β1 were rarer in CP and only occasionally expressed integrin β7. PBs that expressed CCR10 but lacked CCR9 dominated during disease (a median of 68.1%) but were rarer in Rec and HC (47.2% and 20.8%, respectively) whereas cells that lacked both markers were instead more common (44.0% and 73.6%, respectively) (Fig. 2D and E).

IgA is the dominant Ab class in the gut but is also expressed at other mucosal surfaces and in bone marrow [36]. Hence, we determined if there was any difference in expression of homing receptors on PBs depending on whether IgA or other Ab classes were expressed. The median fluorescent index was indeed higher for integrin β7, CCR9, and CCR10 on IgA expressing PBs from CP than in cells expressing other antibody classes (Supplementary Fig. S3C). Accordingly, IgA^+^ PBs more commonly belonged to integrin β7 and CCR9/CCR10 expressing subsets (Fig. 2C and F). However, regardless of IgA expression, PBs from CP most commonly belonged to integrinβ1^high^/integrinβ7^low^ and CCR9^−^/CCR10^+^ populations, suggesting similar homing patterns during early disease for IgM, IgG, and IgA expressing PBs.

### The early COVID-19 plasmablast response targets S and N proteins and is cross-reactive against S of previously encountered coronaviruses

**Figure 3.**
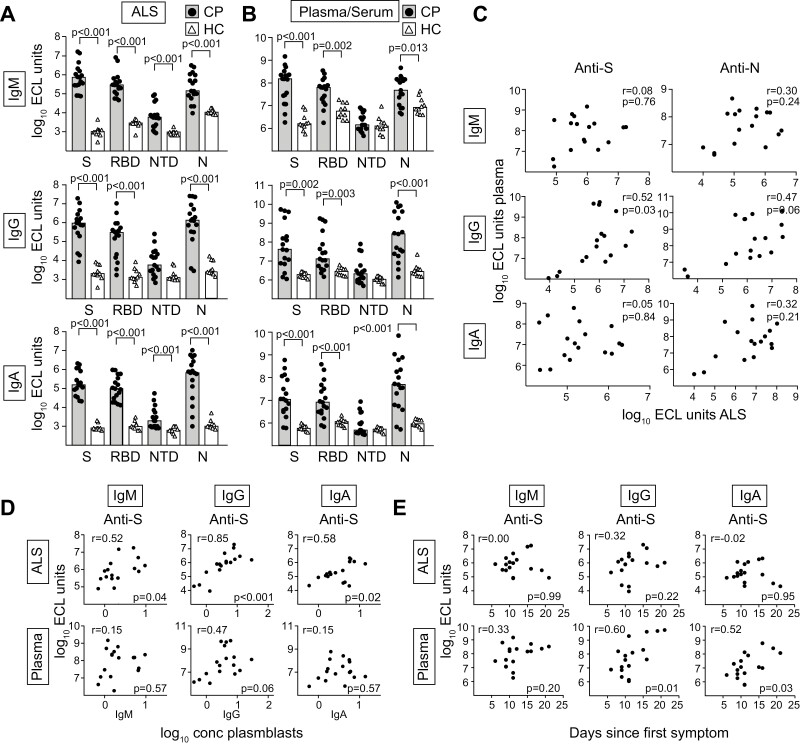
Plasmablasts in COVID-19 patients secrete Abs reactive against the spike and nucleocapsid proteins of SARS-CoV-2. Plasma or Abs in lymphocyte supernatant (ALS) from non-stimulated peripheral blood mononuclear cells from COVID-19 patients (CP; black dots, *n* = 17) or serum and ALS from pre-pandemic control individuals (HC; white triangles, *n* = 10) were prepared and tested for IgM, IgG and IgA reactivity against the spike protein (S), receptor binding domain of the S protein (RBD), the N terminal domain of the S protein (NTD), and the nucleocapsid protein (N) using a multiplex electrochemiluminescence assay. (**A, B**) Reactivity of IgM, IgG, and IgA Abs against the indicated proteins in (A) ALS and (B) plasma/serum from CP and HC. (**C**) Correlation plots that show the reactivity against S and N in plasma versus supernatants for IgM, IgG, and IgA from CP. (**D**) Correlation plots that show Ab reactivity against S in plasma or ALS compared to the concentration of PBs expressing IgM, IgG, and IgA, as determined by flow cytometry. (**E**) Correlation plots that show reactivity against the S protein in ALS and plasma compared to the number of days since first symptoms. Statistical comparisons between groups and correlations were calculated using nonparametric methods (Mann–Whitney with Holm–Bonferroni correction for multiple comparisons, and Spearman rank correlation). Additional data including plasma comparisons between CP and a separate pre-pandemic healthy control group, S + N and N protein reactivity versus PB concentration or time since first symptoms, and correlations in Ab reactivity between antigens can be found in Supplementary Fig. S4.

We next determined which viral antigens were targeted during the early PB response. The reactivity of Abs secreted into lymphocyte culture supernatants (ALS) is a reliable assay to determine PB specificity that correlates well with the concentration of cells detected using ELISPOT [37] that has previously been used extensively as a proxy measurement for human mucosal responses [38–40]. The reactivity of supernatants collected from 17 CP (a subgroup of the 26 used for flow cytometry) was compared to corresponding pre-pandemic samples collected from healthy volunteers using a multiplex ECL assay. PBs from CP produced large amounts of SARS-CoV-2 reactive IgM, IgG, and IgA Abs against both the S and N proteins (Fig. 3A). The levels of IgM and IgA reactivity in ALS against the S protein and the RBD fully separated CP and HC individuals, while some patients did not show increased levels for IgG, suggesting that PBs producing S-reactive IgG formed slightly later during disease than those producing IgA and IgM. A more extensive overlap was evident between groups of individuals when plasma from CP was compared to serum from the HC (Fig. 3B) or plasma from a separate group of pre-pandemic controls (Supplementary Fig. S4A). The levels of Abs targeting SARS-CoV-2 antigens in plasma from CP showed a wide distribution, with levels similar to those observed in HC in some individuals and 1000-fold higher in others.

The levels of Ab reactivity detected in CP ALS did not correlate significantly with plasma Ab levels, except for a relatively weak correlation (*r* = 0.52, *P* = 0.03) for S-reactive IgG Abs (Fig. 3C). There was a strong correlation (*r* = 0.77, *P* = 0.0005) between the total concentration of PBs detected in blood and the combined reactivity against S and N in ALS (Supplementary Fig. S4B). Concentration of PBs expressing IgM, IgG, or IgA showed a rather good correlation with the corresponding class-specific ALS reactivity against both S and N (Fig. 3D, Supplementary Fig. 4C). Plasma levels of S- and N-specific IgG and IgA Abs in CP instead correlated to the number of days since first symptoms (Fig. 3E, Supplementary Fig. S4D). There was a high correlation between S and RBD reactivity for all Ab classes, both for plasma and ALS (*r* = 0.85–0.98, *P* ≤ 0.0001) as well as for reactivity against S and N (*r* = 0.73–0.89, *P* ≤ 0.001) (Supplementary Fig. S4E, S4F, and S4G). There were also significant correlations between Ab classes (*r* = 0.72–0.94, *P* ≤ 0.002), suggesting that most patients generally initiated responses of similar relative strength of all Ab classes (Supplementary Fig. S4H).

**Figure 4.**
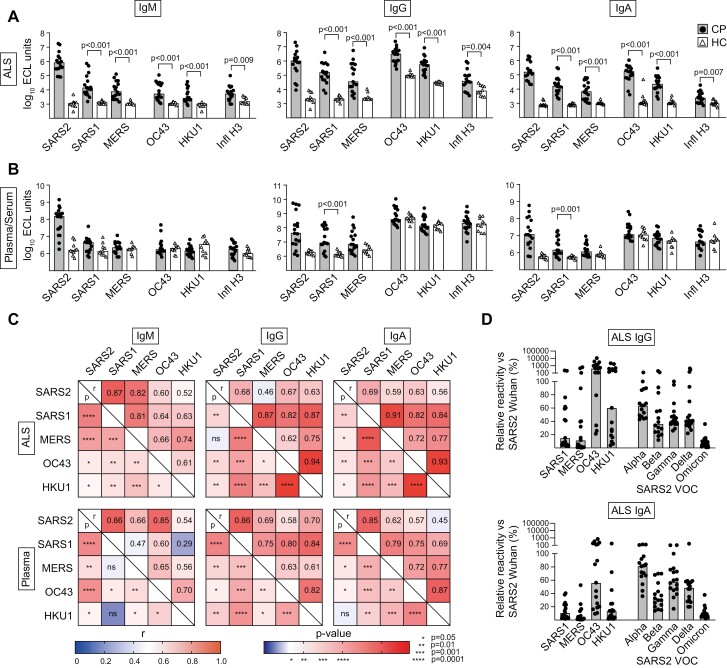
Abs against the spike protein secreted by PBs during the early response to SARS-CoV-2 are cross-reactive to spike protein of other beta-CoVs. Plasma and ALS (for details see Fig. 3) were tested for reactivity against spike (S) proteins from SARS-CoV-1 (SARS1), MERS, OC43, and HKU1 beta-CoVs. Data using the H3 protein from Hong Kong influenza A antigen and SARS-CoV-2 (SARS2; from Fig. 3) are included as references. (**A**) The reactivity of ALS and (**B**) plasma/serum against the proteins are shown. Mann–Whitney with Holm–Bonferroni correction was used to calculate *P* values. (**C**) Matrixes that show the correlation between reactivity for the different S antigens for each Ab isotype for plasma and supernatants from CP. The upper right part of each matrix shows numbers representing *r* values, and the lower left the significance of the correlations. For *r*, values higher than 0.5 are red and below that blue, and for *P*, values higher than 1.3 (10^−1.3^ = 0.05) are red and below that blue. (**D**) Reactivities of IgA and IgG in ALS to S from SARS-CoV-2 variants of concern (VOC) and other beta-CoV relative to the original Wuhan strain are shown.

We next determined the production of Abs reacting against S proteins from other beta-CoVs (SARS-CoV-1, MERS, OC43, and HKU1) as well as the H3 protein from the Hong Kong Influenza A virus, as a control. As expected, levels of Abs reactive against the non-circulating SARS-CoV-1 and MERS viruses were low in ALS or serum from pre-pandemic HC (Fig. 4A and B). Increased reactivity was detected in ALS and plasma from CP, suggesting cross-reactivity of PBs formed in response to SARS-CoV-2 S protein and these related beta-CoVs. In ALS samples, substantially increased reactivity was also detected against the endemic beta-CoVs OC43 and HKU1 when CP and HC were compared. In plasma/serum from both CP and HC, class-switched Ab reactivity against the endemic viruses OC43, HKU, and Influenza was detected, suggesting production initiated after previous infections. However, significant differences were not evident between CP and HC for these beta-CoV strains in plasma/serum at this early timepoint.

A closer comparison of ALS from CP revealed that IgG and IgA reactivity showed a stronger correlation between the different non-SARS-CoV-2 beta-CoV than to SARS-CoV-2 (Fig. 4C). This suggests that the early SARS-CoV-2 PB response is partly generated through activation of pre-existing class-switched memory B cells with cross-reactivity against other beta-CoV and partly through selection of naïve B cells specifically reactive against SARS-CoV-2.

We extended our analysis to also determine whether the PB response targeted SARS-CoV-2 VOC that appeared later during the pandemic. This was indeed the case, with the S protein from Alpha, Beta, Gamma, and Delta variants being targeted by IgG as efficiently as S from HKU1 but not as efficiently as OC43 (Fig. 4D). PB IgA responses to VOC were comparable to those against OC43 and stronger than to other beta-CoVs. However, both for IgG and IgA the Omicron variant stood out, with low PB responses comparable to those targeting SARS-CoV-1 and MERS.

### PB responses can be detected several months after infection, but late plasmablast and memory cells are less cross-reactive towards endemic beta-CoVs than during the early responses

**Figure 5.**
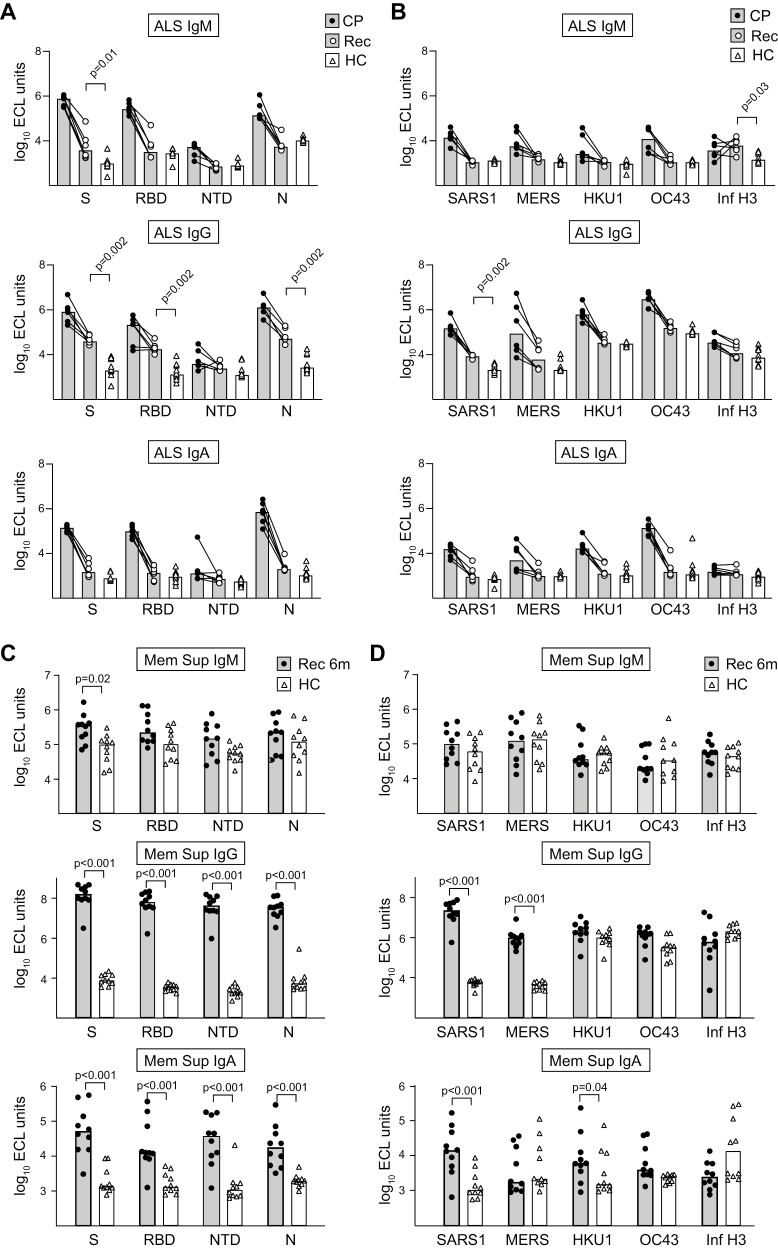
PB IgG responses detectable 3 months after recovery show diminished cross-reactivity, while IgG and IgA memory B cells 6 months after recovery show cross-reactivity to non-circulating but not endemic strains. (**A, B**) ALS from cultures of PBMCs collected three months after recovery (Rec) were analyzed for (**A**) reactivity against the spike protein (S), receptor binding domain of the S protein (RBD), the N terminal domain of the S protein (NTD), and the nucleocapsid protein (N) from SARS-CoV-2 and compared to ALS collected from the same patients when they arrived at hospital (*n* = 6) and to that from non-infected pre-pandemic controls (*n* = 10) or (B) against the spike protein from SARS-CoV-1 (SARS1), MERS, OC43, and HKU1 beta-CoVs as described in Figs. 3 and 4. (**C, D**) Frozen PBMC samples collected from patients 6 months after infection (Rec 6m, *n* = 10) and non-infected controls (HC, *n* = 10) were stimulated with R848 and IL-2 for 9 days and supernatants collected and analyzed as in (A and B). Statistical comparisons between groups were calculated using non-parametric methods (Wilcoxon matched-pair signed rank test for paired [CP vs Rec], Mann–Whitney for non-paired [Rec vs HC]) with Holm-Bonferroni correction for multiple comparisons within each panel; *P* values are only given for significant differences. Paired samples where Ab levels moved in the same direction for all six patients had individual *P*-values of 0.03; these did not reach significance after compensating for multiple testing.

We did not detect increased levels of PBs in blood from patients 2–3 months after disease recovery (Fig. 1, Supplementary Fig. S1). However, some reports have suggested that SARS-CoV-2 PB responses may be rather long-lived, possibly due to ongoing activation of cells in the gut [41, 42]. We did not determine antigen-specificity of PBs using flow cytometry here and it is difficult to determine whether Abs in serum represented ongoing B cell responses or IgG Abs maintained in serum that is not actively produced due to long serum half-life. The analysis of Abs in ALS is a direct sensitive assay for detection of ongoing formation of antigen-specific PBs. Thus, ALS was collected from six of the patients 3 months after their initial infection. As expected, levels of Abs targeting the S and N protein from SARS-CoV-2 in supernatants had decreased at this time in all the patients for all Ab classes (Fig. 5A). Most other beta-CoV also showed less Abs targeting them at this timepoint (Fig. 5B). Nevertheless, the levels were still higher than in HC for some antigens and classes. In particular, IgG expressing PBs that targeted SARS-CoV-2 antigens were still detected after 3 months while less IgA or IgM producers were present at this time point. Interestingly, the lingering PB responses did not display significant cross-reactivity against other beta-CoV (Fig. 5B).

This led us to address whether memory B cells would produce cross-reactive Abs or not. To this end, memory B cells from PBMC samples collected six months after infection were polyclonally stimulated and supernatants collected [43]. While we detected production of IgG Abs targeting SARS-CoV-2 in all individuals, IgA production was more varied and no increases in specific IgM production was detected in CP compared to HC, except for a weakly increased response against the S antigen (Fig. 5C). Interestingly, increased IgG cross-reactivity to SARS-CoV-1 and MERS was detected in cultures from previously infected patients compared to HC. In contrast, relatively high levels of IgG Abs binding to the endemic beta-CoVs HKU and OC43 were detected even in HC and no further increase was detected after infection (Fig. 5D). However, unlike what was observed for the PB response, the cross-reactivity of memory B cells against SARS-CoV-2 VOC was much stronger than to non-related beta-CoV strains (Supplementary Fig. S4I).

### CD45RB^+^CD69^+^ B cells are enriched in blood of COVID-19 patients

**Figure 6.**
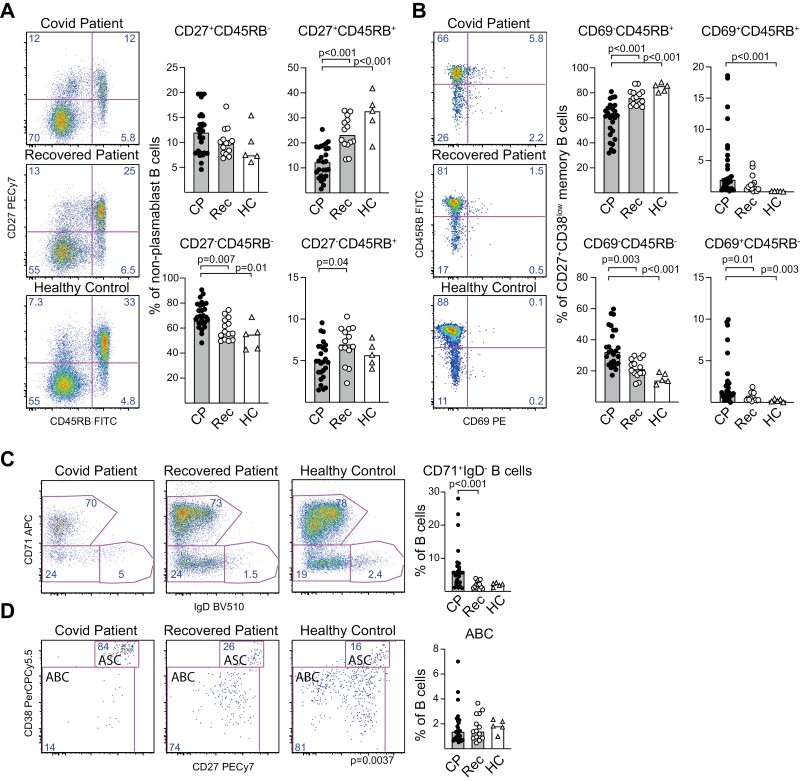
Decreased proportions of B cells in COVID-19 patients have an archetypical memory B-cell phenotype, but increased proportions express CD69. Naïve, memory, and PB CD19^+^ B cells were identified based on CD27 and CD38 expression using flow cytometry. (**A**) The proportions of archetypical CD27^+^CD45RB^+^ memory, CD27 and CD45RB only memory and naïve double negative B cells among non-PB B cells were determined in COVID-19 patients (CP, black dots, *n* = 26), recovered patients (Rec, white dots, *n* = 12) and healthy controls (HC, white triangles, *n* = 5). (**B**) The proportions of CD27^+^CD38^low^ memory B cells that express CD45RB and/or CD69 were determined. (**C**) The proportion of CD71^+^IgD^−^ B cells and (**D**) the proportion of these that are antigen secreting cells (ASC) or activated B cells (ABC). The proportions that were CD71^+^IgD^−^ or CD71^+^IgD^−^CD27^low^CD38^low^ ABC among all B cells were calculated. (A-D). Individual values are marked with black dots and bars indicate group medians. Statistical comparisons between groups and correlations were calculated using non-parametric methods (Mann–Whitney Kruskal–Wallis followed by Dunn’s multiple comparisons test and Spearman rank correlation); only significant differences are indicated. Additional information about gating strategies and controls and further analysis of ABC cells can be found in Supplementary Fig. S5.

Several populations of activated or early memory B cells distinct from PBs and archetypical memory cells have been described in humans [44–46]. Although the number of SARS-CoV-2 specific memory cells may be too low to influence the size of the total memory compartment, the proportion of intermediate cells may increase sufficiently to be detected. One such early memory phenotype is defined by the expression of a CD45RB-specific glyco-epitope in the absence of the canonical memory marker CD27 [45, 47, 48]. The proportion of CD27^−^CD45RB^+^ B cells was not specifically enriched in CP or Rec compared to HC (Fig. 6A). In line with what was described above (Fig. 1B), there was a diminished proportion of B cells that represented archetypical CD27^+^CD45RB^+^ memory cells during disease (Fig. 6A).

CD69 has traditionally been considered an early activation marker, particularly when expressed by T cells [49]. However, CD69 expression is also found in tissue-resident memory T cells, and a recent study that focused on expression of CD69 and CD45RB on memory B cells found that a CD27^+^CD69^+^CD45RB^+^ phenotype was associated with tissue-resident memory B cells [50]. We found a significantly increased proportion of CD27^+^ B cells that expressed CD69 in CP, both among cells positive and negative for CD45RB, while blood from HC largely lacked B cells belonging to these two populations (Fig. 6B).

Limited information is available regarding the expression of Ab classes in CD69^+^ tissue-resident-like B cells in circulation. In CP, the only group in which tissue-resident-like B cells were reliably detected, we found IgM and IgA expression in all CD27^+^ memory B cell populations regardless of their expression of CD45RB or CD69 but found slightly less expression of IgA in CD45RB^+^CD69^+^ compared to CD45RB^+^CD69^-^ cells (Supplementary Fig. S5C).

A population of early circulating memory cells, termed activated B cells (ABC), has been detected in Ebola patients and after Influenza vaccination [44]. They were described to have the same CD19^+^CD71^+^IgD^−^ phenotype as ASC but to be different from these by their distinct expression pattern of CD38 and CD20. Using an identical gating strategy as used when ABC were first described [44], we detected this population in CP (Supplementary Fig. 5A). However, CD27 and CD38 were as efficient as CD20 and CD38 to distinguish ABC from ASC, with the former being IgD^-^CD71^+^CD38^low/−^CD27^low/−^ and the latter IgD^−^CD71^+^CD38^high^CD27^high^ (Supplementary Fig. S5B). In our cohort, we found increased numbers IgD^−^CD71^+^ B cells in CP compared to Rec and HC, but this was only due to an increased number of ASC while the proportions of ABC were similar (Fig. 6C and D). ABC expressed IgM and IgA at levels comparable to other IgD^−^ B cells in CP, Rec, and HC (Supplementary Fig. S5D).

### Increased levels of activated cTfh cells in COVID-19 patients

Expression of CXCR5 gives T cells the potential to enter B-cell follicles to become Tfh cells [51]. Expression of additional activation markers, in particular CD38, ICOS, or PD1, on blood circulating CXCR5^+^ T cells have been shown on cells directly related to Tfh cells in ongoing GC reactions, and an increase in such activated circulating Tfh (cTfh) cells have been suggested as surrogate markers for GC formation after vaccination.

**Figure 7.**
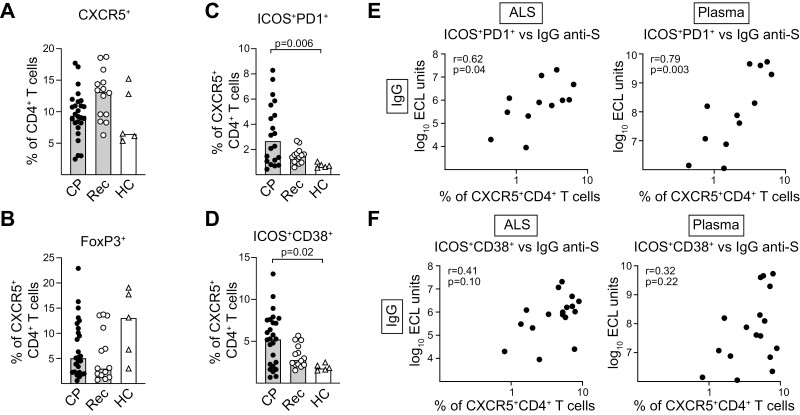
Increased proportions of germinal center-like activated cTfh in COVID-19 patients. (**A**) The proportions of CD4^+^ T cells in COVID-19 patients (CP), recovered patients (Rec), and healthy controls (HC) that express CXCR5. (**B–F**) The proportion of CXCR5^+^ CD4^+^ T cells expressing (B) FoxP3, (C) ICOS and PD1, and (D) ICOS and CD38 in the different groups. For CP *n* = 26 (except in (C) where *n* = 20); Rec *n* = 12; and HC *n* = 5. (A-D) Individual values are marked with symbols and group medians with bars. (E, F) Correlation plots that show IgG reactivity against S in ALS or plasma from CP compared to the proportion of CD4^+^CXCR5^+^ cells in blood that express (E) ICOS and PD1 (*n* = 12) or (F) ICOS and CD38 (*n* = 17). Statistical comparisons between groups and correlations were calculated using nonparametric methods (Mann–Whitney Kruskal–Wallis followed by Dunn’s multiple comparisons test and Spearman rank correlation); only significant differences are indicated. Additional information about gating strategies and controls can be found in Supplementary Fig. S6.

We found that the total proportions of cells that expressed CXCR5 among CD4 T cells in CP were similar to that in Rec or HC, likely reflecting that a relatively low number of new antigen-specific cells had formed (Fig. 7A, Supplementary Fig. S6A). The proportion of T follicular regulatory (Tfr) cells among CXCR5^+^CD4^+^ T cells was also comparable (Fig. 7B, Supplementary Fig. S6B). However, there was an increased proportion of GC-like cTfh cells in CP compared to HC that expressed activation markers (CD38, ICOS, and PD1), suggesting that these derived from ongoing GC responses (Fig. 7C and D, Supplementary Fig. S6C and S6D). The proportion of cTfh that expressed ICOS and PD1 significantly correlated with plasma levels of Abs against SFig. 7EFFig. 7E and N for all Ab classes except IgM against N, while CD38 and ICOS expression did not (Fig. 7E and F, Supplementary Fig. S6G and S6H). The proportion of ICOS^+^PD1^+^ cTfh also correlated significantly with levels of S-specific IgG in ALS (Fig. 7E).

## Discussion

Here we have studied phenotypic markers on and Ab secretion from blood PBs during COVID-19 disease and after recovery in previously immunologically SARS-CoV-2 naïve individuals. Previous studies have mainly characterized the serum Ab response and its specificity, giving insights into targeted epitopes, kinetics, and the strength of the response in relation to disease severity. Still, measurements of serum Abs do not give a fully accurate view of the actual levels of B cell activation since (i) IgA and IgM Abs secreted at mucosal surfaces do not enter the bloodstream, (ii) the half-life of serum Abs differ between isotypes and subclasses, and (iii) differential sequestration of Abs may occur following binding to antigens or Fc receptors. Direct studies of lymphoid organs do address B-cell activation but are hampered by the scarcity of samples derived from living donors and that the complexity of the airway immune system requires multiple inductive sites to be sampled to fully cover the response [36]. Measurements of PBs in the blood stream can act as a proxy for ongoing B-cell activation, as most PBs migrate in blood before they home to effector sites to become mature PC. However, fresh samples are usually required for analysis of PBs as these may not survive cryopreservation as well as other lymphoid cells [52–54]. Here we investigate early B-cell activation and PB formation in fresh blood samples during acute COVID-19 infection and convalescence.

In agreement with previous studies, we found that PB formation was efficient in hospitalized COVID-19 patients [10]. In fact, the levels of PBs in blood were higher than that described for the primary response to an attenuated yellow fever virus vaccine or the recall response to an influenza vaccine, but almost comparable to that observed during acute dengue fever [55]. The response appeared to be mostly made up of cells activated through antigen-specific interactions rather than polyclonal activation, as the PBs produced high levels of IgM, IgG, and IgA Abs binding to SARS-CoV-2 antigens, but little Abs directed against influenza H3. Furthermore, the frequency of PBs and memory B cells expressing different Ab subclasses did not correlate, arguing against unspecific polyclonal activation of memory cells.

Given that the PB response appeared to be antigen-specific, the occurrence of PBs producing Abs that bind S domains from other beta-CoVs most likely represents activation of cross-reactive memory B cells. Importantly, the cross-reactivity was more pronounced for IgA and IgG than for IgM, although most of our patients were tested at early time points when it was unlikely that PB formation was dominated by cells from newly formed GC. This suggests that these early PBs derived from already switched memory cells formed during previous endemic beta-CoV infections. This is in line with that increased levels of Abs reactive against beta-CoV have been detected in serum after COVID-19 [24, 25]. Activation of cross-reactive memory B cells could explain why IgG and IgA SARS-CoV-2 reactive Abs are often detected before or at the same time as IgM Abs in COVID-19 patients [8–10]. This has been suggested previously, based on increased levels of cross-reactive Abs in serum and the presence of SARS-CoV-2 binding memory B cells in SARS-CoV-2 naïve individuals, but our data give more direct support to this hypothesis [24, 25, 30]. As far as we are aware, this is in fact the first direct demonstration of the formation of large numbers of cross-reactive, class-switched PBs early in the response. Nevertheless, we acknowledge that our study is not an ultimate proof for early activation of cross-reactive B cells; for this deep VDJ sequencing of cross-reactive B memory cells in non-infected individuals followed by the detection of PBs derived from the same B cell clones after infection would be needed. However, given that, to our knowledge, such studies have not been performed and that most individuals in the world have now been exposed to SARS-CoV-2 antigens, this may not be possible, but our data further support the hypothesis.

Activation of preexisting memory B cells is a well-known phenomenon when individuals are infected with novel influenza type A strains [27]. Similarly, recent studies of patients infected with SARS-CoV-2 VOC have demonstrated that the response is influenced by vaccinations and infections with previous strains [28, 29]. However, the antigenic distance between endemic beta-CoV viruses and SARS-CoV-2 is likely larger than in these cases, and our study thus supports that this is a general phenomenon. Interestingly, the VOC SARS-CoV-2 strains were targeted even in the very early response, but this was even more pronounced for antibodies produced by memory B cells. This conforms with the notion that cross-reactivity is stronger to more closely related antigens than those more distinct. However, this was not true for the omicron SARS-CoV-2 strain, in particular during the early response, suggesting that the development of this strain may have been driven by an escape from Ab, potentially in an immunodeficient individual, either due to a non-cleared infection or serum treatment.

Before infection, the levels of cross-reactive memory B cells, able to target SARS-CoV-2 and non-circulating non-endemic strains, i.e. SARS-CoV-1 or MERS, appear to be low. In contrast, *in vitro* stimulated memory B cells from non-infected individuals produced high levels of Abs targeting the endemic beta-CoVs OC43 and HKU1 as well as influenza. In addition, after recovery from COVID-19 disease, PBs appeared to shift from cross-reactivity towards SARS-CoV-2 specificity. Thus, it appears that the GC response gradually skewed the binding of Abs from PBs towards less cross-reactivity. Furthermore, the observation that there was no detectable increase in memory B cells reactive to endemic beta-CoV strains after COVID-19 infection, while B cells reactive against the original Wuhan SARS-CoV-2 strains, later developing VOC and the more closely related SARS-CoV-1 and MERS could be detected, indicated that wide cross-reactivity is relatively rare. Nevertheless, we recently found clonal relationships between PBs formed during COVID-19 disease in previously SARS-CoV-2 naïve patients and long-lived memory B cells one year later, although such cells were relatively rare and did in general not show cross-reactivity against other beta-CoV [56]. Further work is needed to understand how previous encounters with coronaviruses influence the likelihood and severity of COVID-19 disease and long-term protection against SARS-CoV-2.

Expression of cellular markers on PBs can give information about where and how the cells were activated and where they will home [11]. We detected IgA, IgG as well as IgM-producing PBs, with IgA1 and IgG1 being the most common subclasses expressed. We found that a large proportion of PBs in the early response expressed CD138, a marker that has been associated with later stages of PB development and/or increased survival of the cells at effector sites [32]. We also found differences in the expression of homing markers on PBs from COVID-19 patients compared to that in healthy controls, with integrin β1 and CCR10 but not CCR9 being expressed on the cells. As far as we are aware, few previous studies have addressed the expression of homing receptors on PBs from COVID-19 patients. Nevertheless, our findings are in line with Sterlin et al. that demonstrated that many PBs expressed CCR10 [10] and Muller et al. that found decreased expression of integrin α4β7 in total T and B cells in patients compared to controls [57]. In addition, we found that CD62L was expressed at relatively high levels in some patients. This pattern is most compatible with a PB response where cells will home to the airways, although high expression of CD62L may also suggest homing to bone marrow or inflamed tissues in at least some patients [11, 36].

This pattern is compatible with a response derived from inductive sites involved in airway immunity, which is not surprising [36]. However, this may not only depend on induction of homing as a consequence of activation in tonsils, inducible bronchus associated lymphoid tissue (iBALT) and mediastinal lymph nodes during the response. Cross-reactive memory B cells formed at these sites during a mucosal priming event as a consequence of an infection with an endemic beta-CoV infection may in fact already be limited to mucosal homing and may even be tissue resident memory cells. For example, after oral priming, memory B cells that form in gut associated lymphoid tissues in mice after an oral priming give mucosal responses even after a systemic boost [58]. Tissue resident memory B cells have indeed been found in both in human gut and mouse lungs [50, 59]. Such tissue-resident memory B cells may explain why SARS-CoV-2 naïve individuals vaccinated intramuscularly with an mRNA-based SARS-CoV-2 vaccine displayed increased levels of IgA Abs in saliva and why this phenomenon was more pronounced after the first than after the second dose [60].

Similar to others, we found that although B-cell concentrations in blood decreased during disease, the T-cell compartment was more severely affected [7]. After recovery, the levels of all B-cell subtypes returned to normal, and we did not find an increase in CD27^−^CD45RB^+^ or CD71^+^IgD^−^ B cells that have been suggested to represent early memory B cells [44, 45, 48]. However, during disease an increased proportion of memory B cells expressed CD69. Non-migratory human gut memory B cells were suggested to have a CD45RB^+^CD69^+^ phenotype, making them similar to CD69^+^ tissue-resident memory T cells [50]. Tissue-resident memory B cells form after influenza infection in mouse lymph nodes and spleen and then migrate to the lungs [59]. Thus, the CD27^+^CD45RB^+^CD69^+^ cells observed here may represent an early wave of memory B cells destined for lung homing to become tissue resident memory cells.

Consistent with previous reports, we found increased numbers of T cells with an activated Tfh like phenotype in COVID-19 patients [61]. Circulating CD4^+^CXCR5^+^ Tfh-like cells originate in lymph nodes and traffic into blood via the thoracic duct in humans [62]. Such cTfh cells display phenotypic, functional as well as clonal and developmental overlap with GC Tfh cells, as supported by epigenetic, transcriptomic and T -cell receptor repertoire studies [62–64]. We observed a correlation between proportions of activated ICOS^+^PD1^+^ cTfh cells and levels of IgG Abs binding to SARS-CoV-2 S antigen in ALS as well as in plasma, supporting that cTfh cells are an interesting and easily accessible biomarker for GC B cell responses even very early during a response. This confirms and extends previous studies primarily focused on cTfh cells during convalescence and their correlation with the neutralizing serum Ab response [61].

Taken together, the data presented here give insights into the early B-cell response during COVID-19 disease. Strengths of the study include the parallel analysis of phenotype, migratory characteristics, and antigen specificity of PBs, the use of fresh samples collected before vaccination or extensive re-infections, the analysis of the specificity against S antigens from several beta-CoVs, the comparisons of PB-produced Abs to both plasma Abs and memory B-cell responses, the relatively large group of patients analyzed during infection, the use of clinically validated flow cytometric assays with clinical reference values set before the SARS-CoV-2 pandemic and analysis of samples during both acute infection and convalescence. Limitations include relatively few convalescence samples included in some of the analyses, that almost all patients studied required hospitalization and the lack of direct flow cytometric assessment of antigen-specific cells.

Collectively, our analyses provide further evidence that the early response to SARS-CoV-2 is dependent on activation of cross-reactive memory B cells originally formed against endemic beta-CoVs. Furthermore, we demonstrate that the response is gradually skewed towards B cells that are less cross-reactive and more specific for SARS-CoV-2. In addition, our analysis of the expression of homing markers suggest that the early response is generated in the upper airways and that the PBs will eventually home to mucosal sites along the airways as well as to the bone marrow.

## Supplementary Material

uxad044_suppl_Supplementary_Material

## Data Availability

The data underlying this article will be shared on reasonable requests to the corresponding authors.

## Abbreviations

Ab: Antibody
ABC: Activated B cells
Abs: Antibodies
ACE-2: Angiotensin converting enzyme-2
ALS: Abs secreted into lymphocyte culture supernatants
ASC: Antibody secreting cells
beta-CoV: Betacoronavirus
CP: Covid Patients
cTfh: Circulating T follicular helper
ELC: Electrochemiluminescence
GC: Germinal center
HC: Healthy controls
iBALT: Inducible bronchus associated lymphoid tissue
N: Nucleocapsid
NTD: N terminal domain
PBs: Plasmablasts
PCs: Plasma cells
RBD: Receptor binding domain
Rec: Recovered Patients
S: Spike
Tfh: T follicular helper
Tfr: T follicular repressor
VOC: Variants of concerns.

**Standard abbreviations (not defined in text):**
CCR10: C-C motif chemokine receptor 10
CCR9: C-C motif chemokine receptor 9
CD: Cluster of differentiation
COVID-19: Coronavirus disease 2019
CXCR4: C-X-C motif chemokine receptor 4
CXCR5: C-X-C motif chemokine receptor 5
DMSO: Dimethyl sulfoxide
EDTA: Ethylenediaminetetraacetic acid
ELISPOT: Enzyme-linked immunosorbent spot
ICOS: Inducible T-cell costimulatory
IgA: Immunoglobulin A
IgD: Immunoglobulin D
IgG: Immunoglobulin G
IgM: Immunoglobulin M
IgA1: Immunoglobulin A1
IgA2: Immunoglobulin A2
IgG1: Immunoglobulin G1
IgG2: Immunoglobulin G2
MERS: Middle East respiratory syndrome–related coronavirus
PCR: Polymerase chain reaction
PD1: Programmed cell death protein 1
PBMC: Peripheral blood mononuclear cells
PBS: Phosphate-buffered saline
RPMI: Roswell Park Memorial Institute
SARS-CoV-1: Severe acute respiratory syndrome virus 1
SARS-CoV-2: Severe acute respiratory syndrome virus 2.
