# Supplementary material for: Plasmablasts in previously immunologically naïve COVID-19 patients express markers indicating mucosal homing and secrete antibodies cross-reacting with SARS-CoV-2 variants and other beta-coronaviruses
Source: Clin Exp Immunol. 2023 Apr 18;213(2):173–89. doi: 10.1093/cei/uxad044 (PMC10361743; doi:10.1093/cei/uxad044)

Supplemental Table 1 – Antibody panel for clinical analysis of cells

Panels used for clinical flow cytometry

**TBNK (BD 662967)**	**B cell panel**
CD3-FITC	IgM-BB515 (BD 564622)
CD16+56-PE	CD24-PE (BD 560991)
CD45-PerCPCy5.5	CD19-PerCPCy5.5 (BD 561295)
CD4-PECy7	CD27-PECy7 (BD 560609)
CD19-APC	CD38-APC (BD 345807)
CD8-APCCy7	CD20-APCH7 (BD 560176)
	CD21-BV421 (BD 562956)
	IgD-BV510 (BD 561490)

Supplemental Table 2 – Antibodies used for extended flow cytometric panel

	**B panel 1**	**B panel 2**	**B panel 3**	**Tfh panel**
FITC	IgG2+IgG3*	Integrin β1-BB515 (BD 564556)	CD45RB (Exbio 1F-224-T100)	CXCR5 (BD 564625)
PE	IgG1+IgG2*	CCR9 (BD 561607)	CD69 (BD 560968)	FoxP3**
PerCPCy5.5	IgA1+IgA2*	CCR10 (BD 564772)	CD38 (BD 551400)	
PECy7	CD27 (BD 560609)	CD27 (BD 560609)	CD27 (BD 560609)	
APC	IgA1+IgG4*	Integrin β7 (BD 551082)	CD71 (BD 551374)	CD3 (BD 561804)
APCR700	CD19 (BD 564977)	CD19 (BD 564977)	CD19 (BD 564977)	CD4 (BD 564976)
APCH7	IgD (BD 561305)	CD20 (BD 560734)	IgA-APCVio770 (Miltenyi 130-113-999)	
BV421	CD138 (BD 565943)	CD62L (BD 563862)	CXCR4 (BD 566282)	ICOS (BD 562901)
BV510	CD38 (BD 563251)	CD38 (BD 563251)	IgD (BD 563034)	CD38 (BD 563251)
BV605	IgM* (BD 562977)	CD138 (BD 563294)	IgM (BD 562977)	PD1 (BD 563245)

5µl of BD Biosciences Brilliant Stain Buffer Plus was added to each stain reaction

* These Abs were used both for extracellular and intracellular staining of cells. IgA and IgG Abs from Cytognos IgH-isotype panel (CYT-IGS-1). Abs were reconstituted as described and 5µl was used for both the extracellular and the intracellular staining.

** FoxP3 Abs from Thermo Fisher Scientific was included in the eBioscience Human Regulatory T Cell Whole Blood Staining Kit (88-8996) and was used as described by the manufacturer.

Supplemental Table 3 – Antigens used for electrochemiluminescence analysis of antibodies using the Mesoscale discovery platform

**Corona Virus panel 1**		**SARS-CoV-2 panel 23**	
SARS-CoV-2 nucleocapsid	Full length	SARS-CoV-2 Spike	Wuhan
SARS-CoV-2 N-terminal	Q14-L303	SARS-CoV-2 Spike (AY.4.2)	Delta
SARS-CoV-2 RBD	R319-F541	SARS-CoV-2 Spike (B.1.1.7)	Alpha
SARS-CoV-2 Spike	Soluble ectodomain	SARS-CoV-2 Spike (B.1.1.529; BA.1, BA.1.15)	Omicron
SARS-CoV-1 Spike	Soluble ectodomain	SARS-CoV-2 Spike (B.1.351)	Beta
MERS-CoV Spike	Soluble ectodomain	SARS-CoV-2 Spike (P.1)	Gamma
HCoV-HKU1 Spike	Soluble ectodomain	BSA	Full length
HCoV-OC43 Spike	Soluble ectodomain		
Flu A/Hong Kong H3	Soluble ectodomain		
BSA	Full length		

Supplemental Figure legends

SUPPLEMENTAL FIGURE 1 Study overview

(A) 49 SARS-CoV-2 infected patients were included in this study. The concentrations of lymphoid cells (TBNK assay with Trucount beads) and B cell subtypes were determined when the patients were acutely sick or 2-3 months after recovery (n=20). Concentration of cells in blood was compared to clinically used normal values established before the pandemic to include values from 95% of healthy blood donors. The proportion of B cells belonging to different subtypes were compared to data collected before the SARS-CoV-2 pandemic from 61 healthy blood donors. (B) In a subgroup of 18 to 26 acutely sick and 11 to 14 recovered patients (exact numbers varied due to limited availability of Abs for the different panels) from the original group, a more detailed phenotypic analysis of B cells and circulating T follicular helper (cTfh) cells was performed and results were compared to those from 5 healthy controls that had not tested positive for SARS-CoV-2 in PCR and did not carry Abs against SARS-CoV-2 until sample collection in early 2020. (C) Abs in plasma samples and lymphocyte supernatants (ALS) were analyzed in a subgroup of 17 of the patients with active disease and 6 of the same patients after recovery. Serum and ALS samples collected from a group of 10 healthy volunteers collected before the pandemic were used as controls. Frozen PBMC samples were prepared 6 months after disease from 10 of the patients in this study and from 10 health care workers that had not experienced any infections, tested positive for SARS-CoV-2 in PCR and did not carry Abs against SARS-CoV-2. After thawing, the PBMCs were stimulated with R848 and IL-2 for 9 days after which cell supernatants were collected. Plasma, serum, ALS and memory stimulation supernatant samples were analyzed for IgM, IgG and IgA Ab reactivity against different antigens from SARS-CoV-2 and other beta-CoVs using a multiplexed electrochemiluminescence method. All statistical analyses have been performed using non-parametric methods.

SUPPLEMENTAL FIGURE 2

(A) The concentration of the indicated cell types was determined in acute COVID-19 patients (CP; n=49) and 2-3 months after recovery (Rec; n=20) using a commercial clinical flow cytometric assay (TBNK assay with Trucount beads; BD Biosciences). Individual patients are indicated with black and white dots and medians within groups with grey bars. Normal ranges comprising 95% of pre-pandemic healthy blood donors are indicated with dashed red lines. (B) Gating strategy for a clinical assay that determines the proportion of B cells belonging to the indicated subtypes. (C) Concentration of different B cell subtypes based on the concentration of B cells and the relative proportion of subtypes (B). Data presented as in (A) with all values rounded to the nearest 0.01 x 10^6 cells. For PBs, a value of 0.01 x 10^6 cells/ml or above is outside the normal range and no lower threshold exists. (D) Gating strategy to determine the proportion of memory B cells and PBs that express the indicated Ab isotypes and subclasses. (E) Staple diagrams indicating the proportion of PBs and memory B cells that express the indicated Ab isotypes and subclasses in individual COVID-19 patients during disease and recovery. Each bar represents an individual patient. Statistical p-values between groups were calculated using Mann-Whitney, and only significant differences ($p<0.05$) are indicated.

SUPPLEMENTAL FIGURE 3

Expression of homing markers on PB. (A) The gating strategy used to define T cells and Naïve, Memory and PB B cells is shown uppermost with controls for the identity of the cells based on expression of CD138 and CD20 below. (B) Median fluorescence values for the expression of the homing markers Integrin β1, Integrin β7, CCR9, CCR10, CD62L and CXCR4 in the indicated cell subtypes in COVID-19 patients (n=18). (C) Median expression of Integrin β1, Integrin β7, CCR9 and CCR10 in IgA^- and IgA^+ PBs in COVID-19 patients (n=12). (D) FMO controls for the gates used in Figure 2 and Supplemental figure 3 are shown. (E) Staple diagrams indicating the relative proportion of PBs falling into the quadrants defined by expression of Integrin β1/Integrin β7 (top) or CCR9/CCR10 (below) in COVID-19 patients during disease (CP), recovered patients (Rec) and healthy controls (HC). Each bar represents an individual patient. Statistical p-values between groups were calculated using Mann-Whitney or Kruskal-Wallis followed by Dunn´s multiple comparison. Only significant differences ($p<0.05$) are indicated.

SUPPLEMENTAL FIGURE 4

(A) Further control for figure 3B showing a comparison of Abs against the spike (S), the receptor binding domain of S (RBD), N terminal domain of the spike (NTD) or the nucleocapsid (N) proteins of SARS-CoV-2 in plasma from COVID-19 patients during disease (CP; marked with black dots) and healthy controls (HC; marked with white triangles). CP plasma samples are the same as in Figures 3 and 4, whereas HC samples (n=9) are from from a separate control group from which plasma instead of serum was collected before the SARS-CoV-2 pandemic. (B) Correlation plots from COVID-19 patients showing total spike (S) plus nucleocapsid (N) Abs in ALS (top) or plasma (plasma) versus the concentration of PBs in blood. (C) Correlation plots showing IgM, IgG and IgA Abs binding to the N protein in ALS (top) or plasma (bottom) versus the concentration of PBs in blood expressing the corresponding Ab isotype. (D) Correlation plots showing binding to the N protein in ALS (top) or plasma (bottom) versus the number of days since first symptoms. (E, F) Correlation plots showing IgM, IgG and IgA Ab reactivity to RBD versus S (E) and N versus S (F) in ALS (top) and plasma (bottom). (G) Correlation matrix for IgM, IgG and IgA Abs reacting binding different SARS-CoV-2 antigens in ALS and plasma samples from COVID-19 patients with the upper right area showing r values and the lower left p values. R values higher than 0.5 are red, and p values higher than 1.3 ($10^{-1.3} = 0.05$) are red. (H) Correlation matrix for the different Abs binding S and N in ALS and plasma samples from COVID-19 patients presented as in (G). (I) Frozen PBMC samples collected from patients six months after infection (n=10) were stimulated with R848 and IL-2 for 9 days and collected supernatants were analyzed for IgG Abs binding to S from SARS-CoV-2 variants of concern (VOC) and other beta-CoVs relative to the original Wuhan strain. (A-H) Mann-Whitney with Holms-Bonferroni correction and Spearman rank correlation was used for statistical calculations. In (A) only significant differences ($p<0.05$) are indicated.

SUPPLEMENTAL FIGURE 5

(A) The gating strategy to determine the proportion of Ab secreting cells (ASC) or activated B cells (ABC) in COVID-19 patients is shown at the top, with a control at the bottom showing that PBs identified based on CD27 and CD38 expression fall into the ASC gate. (B) Example plots demonstrating that diving $CD71^{+}IgD^{-}$ cells into $CD27^{high}CD38^{high}$ ASC and $CD38^{low/-}$ $CD27^{low/-}$ ABC gives the same result as gating based on CD38 and CD20 (as originally described [44]). To the left the expression of CD38 and CD20 on all $CD71^{+}IgD^{-}$ B cells is shown, in the middle on ACS (defined as $CD71^{+}IgD^{-}CD27^{high}CD38^{high}$ cells) and to the right on ABC (defined as $CD71^{+}IgD^{-}CD38^{low/-}CD27^{low/-}$ cells). (C) Representative dot plots showing the number of cells expressing IgA or IgM among $CD27^{+}$ memory B cells from COVID-19 patients (CP) divided into subgroups based on expression of CD45RB and CD69 and (D) in ABC (top) and $CD19^{+}CD71^{-}IgD^{-}$ B cells (bottom). To the right of the dot plots in (C) the proportions of IgM (top) and IgA (bottom) expressing cells are shown in the indicated subgroups in CP, and to the right of the dot plots in (D) the proportion of IgA (left) and IgM (right) expressing cells are shown in CP, recovered patients (Rec) and healthy controls (HC). Statistical p-values between groups were calculated using Kruskal-Wallis followed by Dunn´s multiple comparison. Only significant differences ($p<0.05$) are indicated.

SUPPLEMENTAL FIGURE 6

(A) Gating strategy to identify $CXCR5^{+}$ CD4 circulating Tfh (cTfh) cells and detection of activation markers (ICOS, PD1 and CD38) and Treg lineage marker (FoxP3) on them. (B,C) FMO controls for gates used to define positive cells. (D) The dot plot to the left shows the expression of ICOS and CD38 on cTfh, the one in the middle expression of ICOS and PD1 on the same cells and the one to the right the expression of ICOS and PD1 on $ICOS^{+}CD38^{+}$ cells. (E, F) Correlation plots comparing the reactivity of the indicated Ab isotypes in ALS and plasma against (E) S and (F) N protein versus the proportion of $ICOS^{+}PD\text{-}1^{+}$ cells among cTfh in blood. Spearman rank correlation was used to calculate statistics.

Supplemental Figure 1

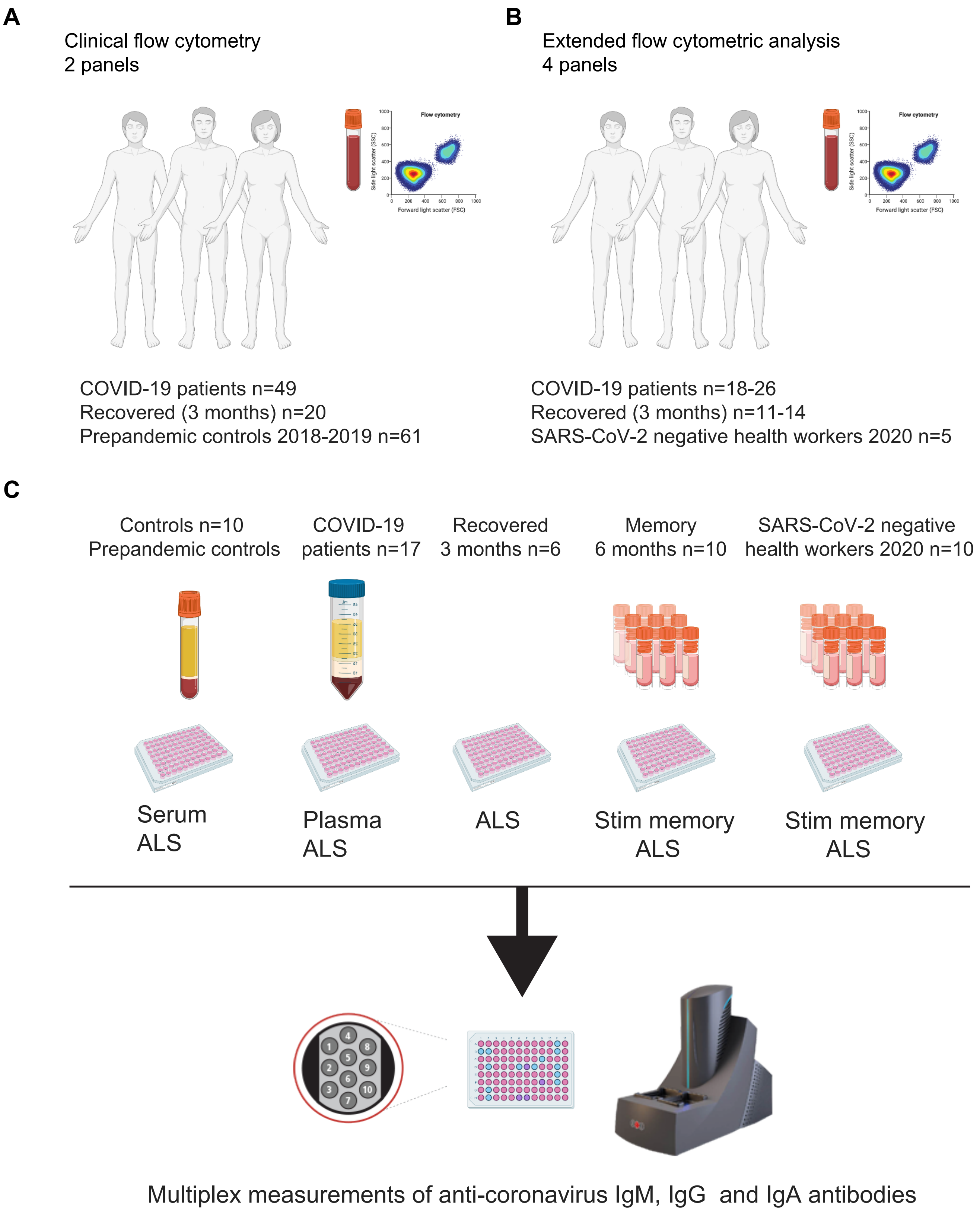

Supplemental Figure 2

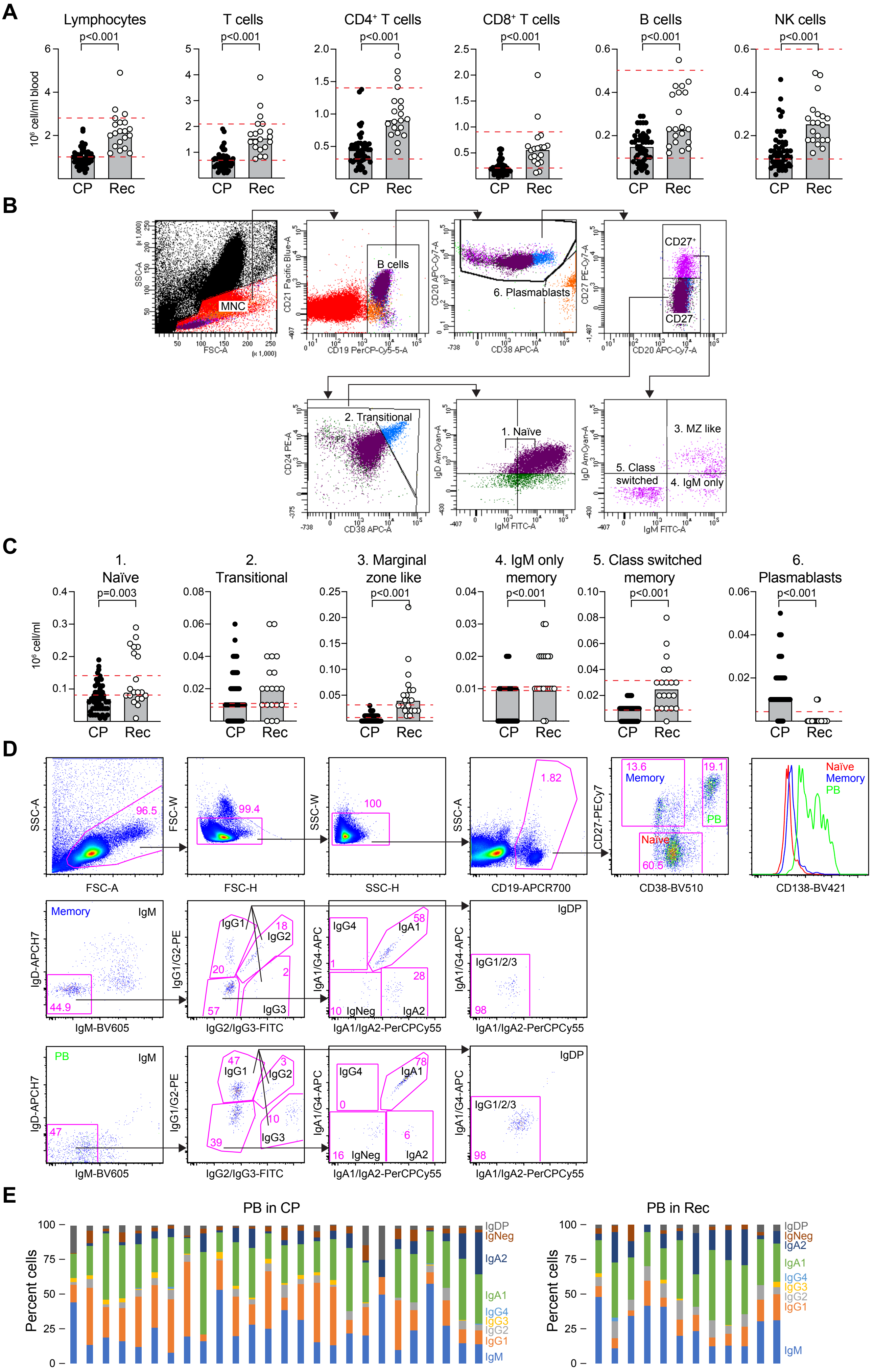

Supplemental Figure 3

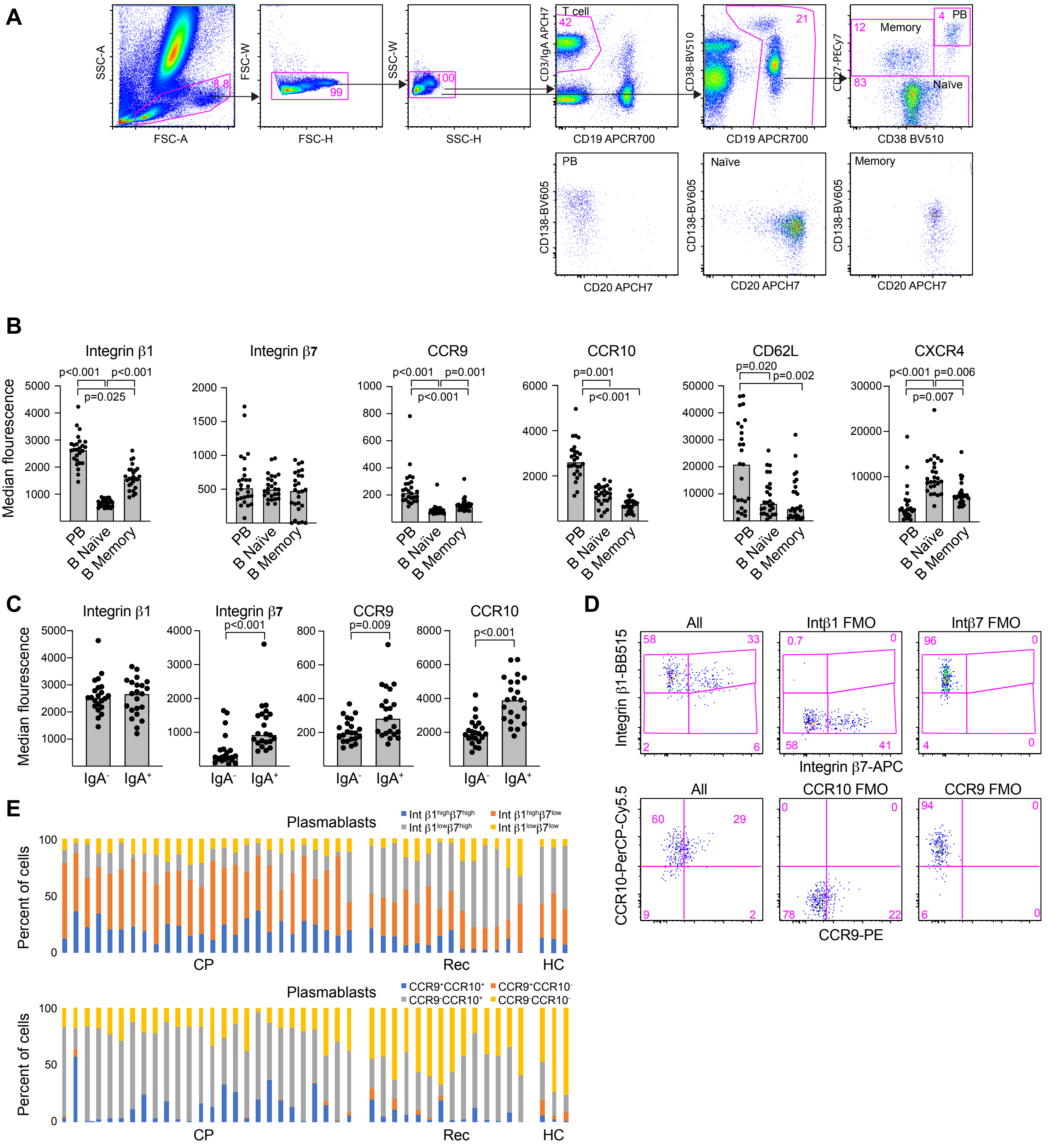

Supplemental Figure 4

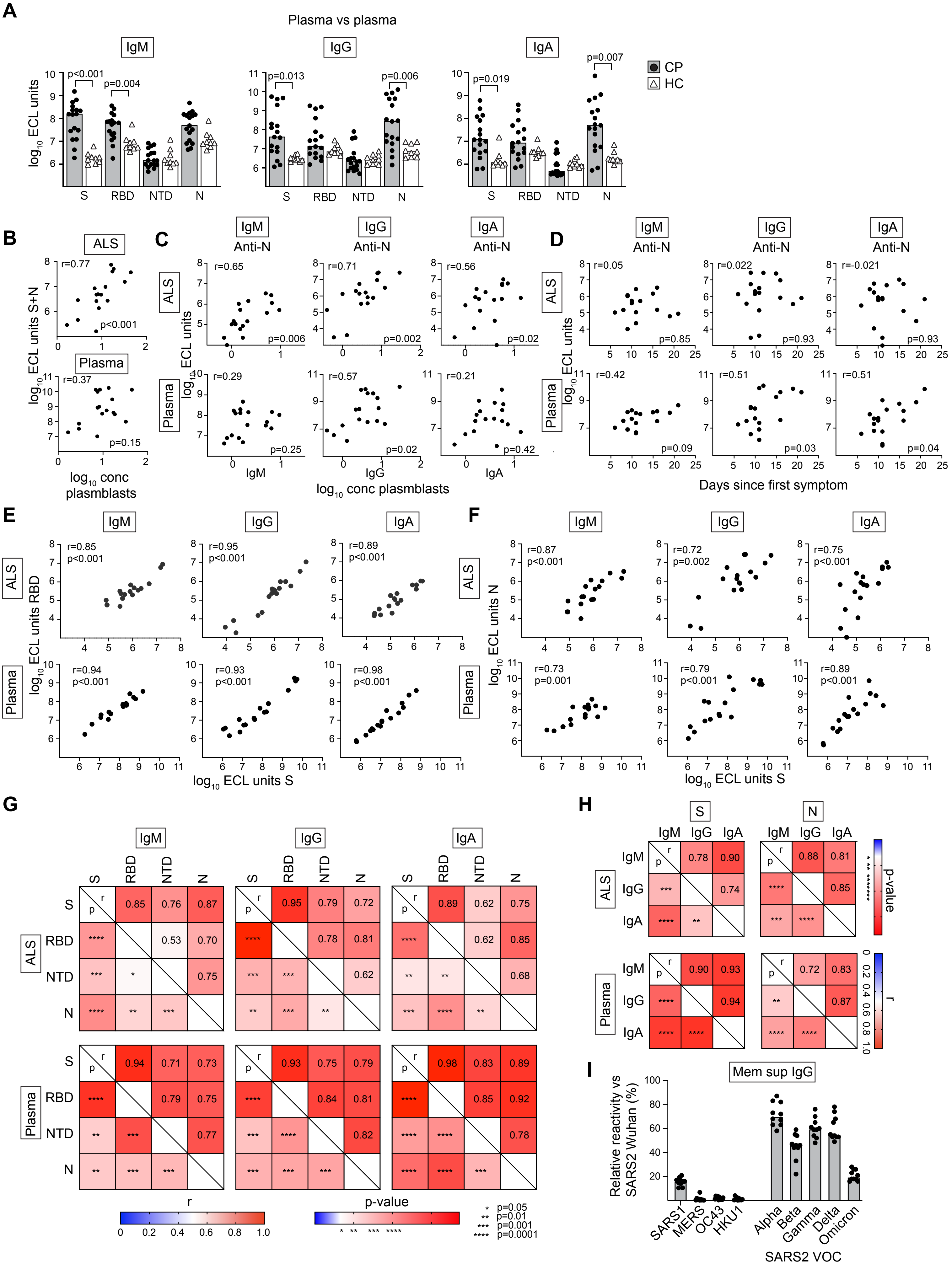

Supplemental Figure 5

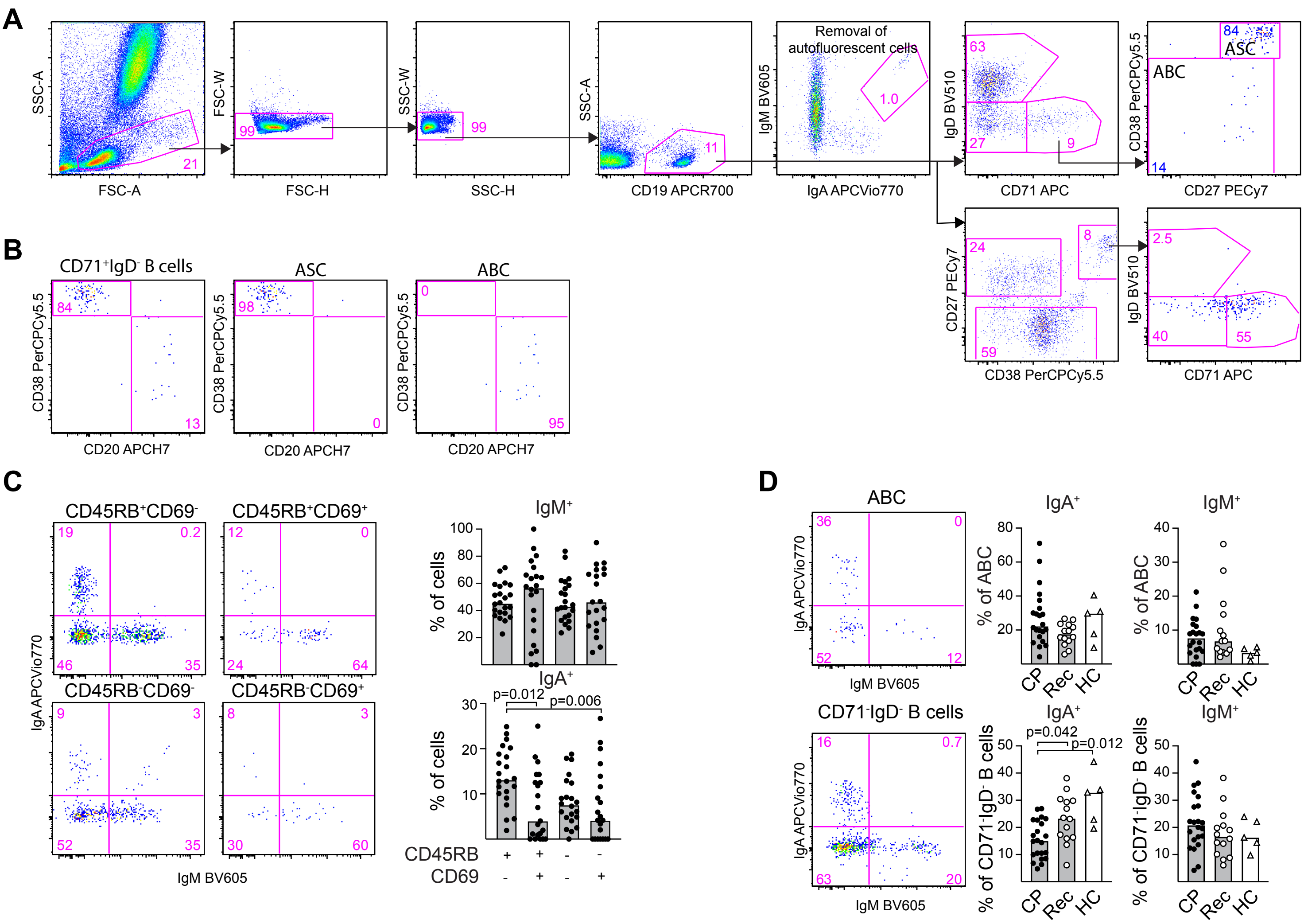

Supplemental Figure 6

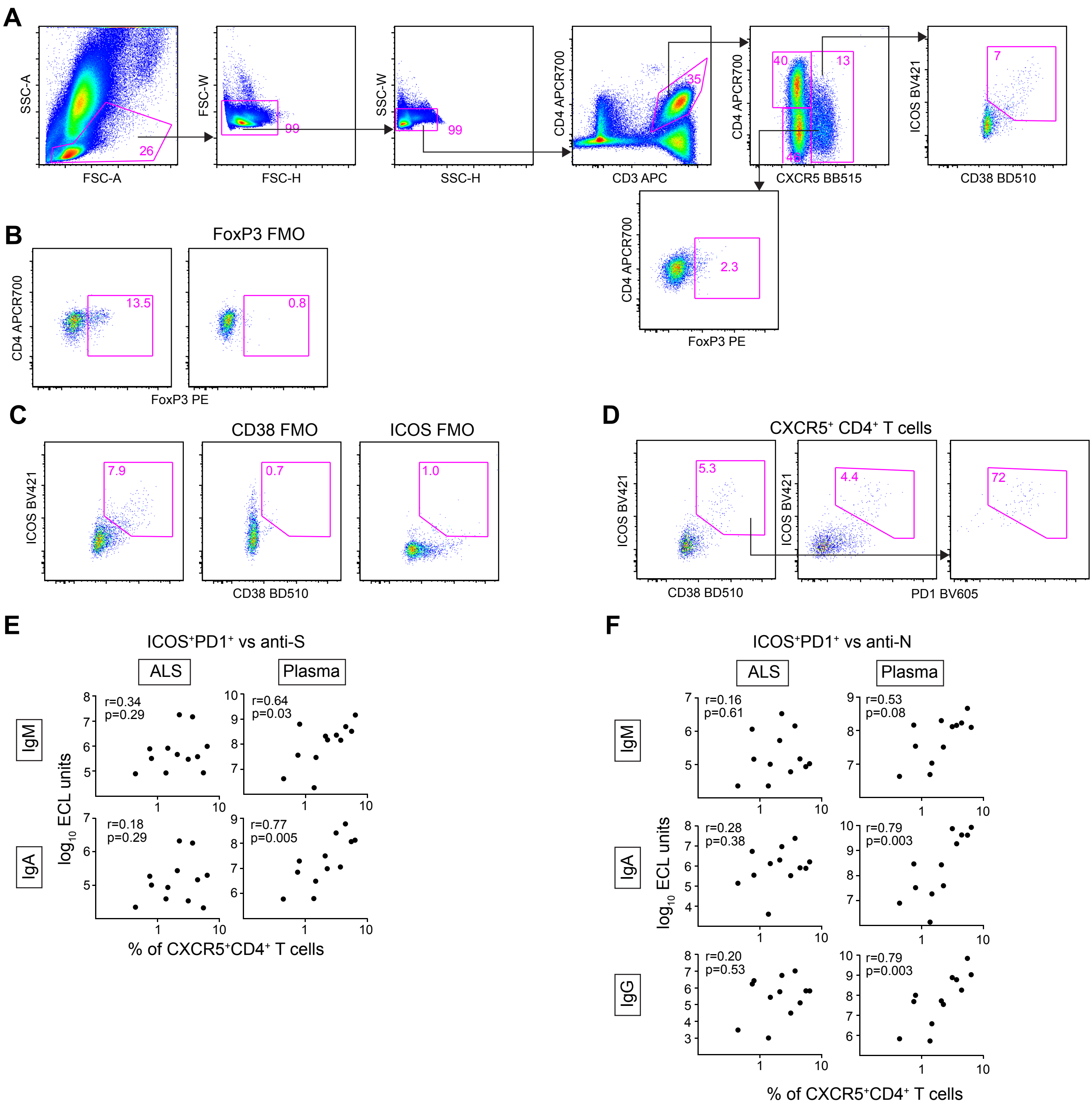

Supplement: uxad044_suppl_Supplementary_Material [file uxad044_suppl_supplementary_material.pdf]
